# Breathing Patterns Indicate Cost of Exercise During Diving and Response to Experimental Sound Exposures in Long-Finned Pilot Whales

**DOI:** 10.3389/fphys.2018.01462

**Published:** 2018-10-25

**Authors:** Saana Isojunno, Kagari Aoki, Charlotte Curé, Petter Helgevold Kvadsheim, Patrick James O’Malley Miller

**Affiliations:** ^1^Sea Mammal Research Unit, Scottish Oceans Institute, School of Biology, University of St Andrews, St Andrews, United Kingdom; ^2^Atmosphere and Ocean Research Institute, The University of Tokyo, Chiba, Japan; ^3^Cerema-Ifsttar, UMRAE, Strasbourg, France; ^4^Norwegian Defence Research Establishment, Horten, Norway

**Keywords:** aerobic diving limit, anthropogenic noise, code:R, DTAG, field metabolic rate, *Globicephala melas*, respiratory rate, sonar

## Abstract

Air-breathing marine predators that target sub-surface prey have to balance the energetic benefit of foraging against the time, energetic and physiological costs of diving. Here we use on-animal data loggers to assess whether such trade-offs can be revealed by the breathing rates (BR) and timing of breaths in long-finned pilot whales (*Globicephela melas*). We used the period immediately following foraging dives in particular, for which respiratory behavior can be expected to be optimized for gas exchange. Breath times and fluke strokes were detected using onboard sensors (pressure, 3-axis acceleration) attached to animals using suction cups. The number and timing of breaths were quantified in non-linear mixed models that incorporated serial correlation and individual as a random effect. We found that pilot whales increased their BR in the 5–10 min period prior to, and immediately following, dives that exceeded 31 m depth. While pre-dive BRs did not vary with dive duration, the initial post-dive BR was linearly correlated with duration of >2 min dives, with BR then declining exponentially. Apparent net diving costs were 1.7 (SE 0.2) breaths per min of diving (post-dive number of breaths, above pre-dive breathing rate unrelated to dive recovery). Every fluke stroke was estimated to cost 0.086 breaths, which amounted to 80–90% average contribution of locomotion to the net diving costs. After accounting for fluke stroke rate, individuals in the small body size class took a greater number of breaths per diving minute. Individuals reduced their breathing rate (from the rate expected by diving behavior) by 13–16% during playbacks of killer whale sounds and their first exposure to 1–2 kHz naval sonar, indicating similar responses to interspecific competitor/predator and anthropogenic sounds. Although we cannot rule out individuals increasing their per-breath O_2_ uptake to match metabolic demand, our results suggest that behavioral responses to experimental sound exposures were not associated with increased metabolic rates in a stress response, but metabolic rates instead appear to decrease. Our results support the hypothesis that maximal performance leads to predictable (optimized) breathing patterns, which combined with further physiological measurements could improve proxies of field metabolic rates and per-stroke energy costs from animal-borne behavior data.

## Introduction

Energy is a key currency that can determine both the cost-benefit and availability of behavioral options to an individual. Animals require energy to survive, grow and reproduce in different stages of their life history in order for their genes to contribute to populations (fitness). To satisfy these energy requirements in varying ecological conditions, animals have evolved physical and behavioral adaptations that enable energy efficient living, as well as acquiring and storing of energy. An individual’s energy requirement is the sum of energy consumption during rest (basal metabolic rate [BMR]), plus the additional cost during routine activities (e.g., digestion, active thermoregulation) plus additional costs during locomotion/exercise (Kleiber, 1961). For slowly evolving species that often rely on behavioral flexibility to cope with environmental variability such as marine mammals (e.g., Pinsky et al., 2010), quantifying the energetic costs of behavior can help to elucidate the mechanisms that drive impacts of environmental change, including anthropogenic stressors.

Field metabolic rates (FMR) are challenging to measure in free-ranging animals that do not easily lend themselves for capture and direct measurement of oxygen consumption. Therefore, proxies for metabolic cost have been used as indicators of energy expenditure; e.g., breathing rates related to cost-of-transport (COT) (Williams and Noren, 2009; Christiansen et al., 2014; Roos et al., 2016), body acceleration (Jeanniard-du-Dot et al., 2016) and fluke stroke rate (Williams et al., 2017). However, the use of such proxies requires careful consideration of the underlying physiology and species-specific calibration. E.g., breathing rate is the number of breaths taken per unit time, but it doesn’t necessarily quantify variations in metabolic rate because tidal volume (VT) and oxygen extraction per breath (ΔO_2_ = inspired-expired O_2_) are not always constant (Fahlman et al., 2016; Roos et al., 2016). Breathing rate can be expected to correlate with mass-specific metabolic rate, assuming tidal volumes scale isometrically with body mass (Mortola, 2015); however, at fine temporal scales, the signal can be masked by variation both in VT or ΔO_2_ (Fahlman et al., 2016, 2017). After a long period of breath hold (dive), oxygen levels in the blood might be reduced and the first few breaths will then result in higher O_2_ extraction rate because the gradient between the alveolar air and the blood is high. Indeed, approaches that account for the effect of breath timing on ΔO_2_ can improve predictions of how O_2_ consumption relates to locomotion effort (Roos et al., 2016). Both ΔO_2_ and VT are expected to vary with activity level and following diving (Fahlman et al., 2017); both VT and ΔO_2_ decrease as a function of time and breath number since a dive (Fahlman et al., 2016). However, respiratory behavior and gas exchange can be expected to be optimized when an animal maximizes physiological recovery to re-establish homeostasis and/or maximize foraging performance, such as after intense exercise (e.g., post-exercise oxygen consumption in humans, Børsheim and Bahr, 2003) or a long-duration breath-hold dive of a marine mammal.

Air-breathing marine predators that target sub-surface prey have to balance the energetic benefit of foraging against the time, energetic and physiological costs of diving (Boyd, 1997; Kooyman and Ponganis, 1998). This makes them central-place foragers that must return to surface (“central place”) to recover depleted oxygen (O_2_) and eliminate excess carbon dioxide (CO_2_) (Boyd, 1997; Kooyman and Ponganis, 1998; Boutilier et al., 2001). A suite of morphological, physiological and behavioral adaptations of breath-hold divers demonstrate strong selection pressure for energetic efficiency and capacity to store both O_2_ and CO_2_ in the body (Ponganis, 2015). Thus, how divers spend time and locomotion effort at depth vs. recovering at surface can help us understand the energetic costs of their foraging decisions.

Body size is a key determinant of diving capacity: larger bodies allow for correspondingly increased storage of O_2_ and CO_2_ in tissues (for a given a body shape and composition), while the mass-specific metabolic rate decreases with body mass (Kleiber, 1961). Thus, one can expect smaller divers to deplete their oxygen stores faster during diving, and larger divers to have lower mass-specific locomotion costs for a given speed (Williams, 1999). However while comparative studies have demonstrated the importance of body size and composition to diving capacity across breath-hold diving species (e.g., Ponganis, 2015), fewer studies have addressed intra-specific variation in the energetic costs of foraging dives due to body size (although see Horning, 2012; Carter et al., 2017), despite its potential importance for stage-specific behavior and survival.

Toothed whales such as pilot whales may be particularly vulnerable to anthropogenic noise pollution as they use sound both to search for food (echolocation signals) and to maintain social contact with conspecifics (Southall et al., 2007). Several studies showed that toothed whales trade-off fitness-enhancing activities in response to anthropogenic noise and to natural threatening stimuli such as predator sounds (e.g., sperm whales, Curé et al., 2016; Isojunno et al., 2016), which can translate to energetic costs due to lost foraging opportunities (e.g., Williams et al., 2006), and physiologically expensive flight responses (e.g., beaked whales, Williams et al., 2017; minke whales, Kvadsheim et al., 2017; pilot whales, Bowers et al., 2018). Such effects may be especially significant when the animal has limited flexibility to compensate for changes in their energy budgets, such as calves, lactating females, or species with high energy requirements relative to prey availability. Long-finned pilot whales and a closely related but more temperate congener species, short-finned pilot whales, have been suggested to employ a “spend more, gain more” strategy where high quality prey demand high foraging costs (Aguilar Soto et al., 2008; Velten et al., 2013; Aoki et al., 2017). Female pilot whales incur additional energetic costs during gestation (12 months) and lactation (2–3 years), during which their energy consumption can increase by 32–63% (Lockyer, 2007). Both species feed mainly on squid (Gannon et al., 1997; Mintzer et al., 2008) and make deep foraging dives (>500 m) (Baird et al., 2002; Aguilar Soto et al., 2008). Therefore, they represent particularly interesting model species to quantify the energetic cost of disturbance.

We used animal-borne sound and movement data loggers to identify breath times and fluke strokes in 17 free-ranging long-finned pilot whales, and analyzed their diving and near-surface behavior to: (1) examine whether breathing behavior immediately before and after long-duration dives can be used to indicate recovery time and energy cost of diving, (2) test for individual differences in post-dive recovery time and breathing rate, in particular whether elevated levels matched expected higher mass-specific metabolic rates for individuals with smaller body size, or potentially lactating individuals (those closely associated with a calf), and (3) quantify any changes in breathing behavior in response to sonar and killer whale (potential predator or competitor) sound exposures, given the diving context. If behavior changes during sound exposures were associated with a stress response, we expected a change in breathing behavior that would not be explained by recovery from previous breath-hold duration or stroking effort.

## Materials and Methods

### Data Collection

Data were collected from 17 long-finned pilot whales (*Globicephala melas*) tagged with audio and movement-recording data loggers using suction cups (DTAG; Johnson et al., 2009). The whales were tagged within the Vestfjord basin off Lofoten in northern Norway (66°–70° N latitude) during the spring and summer 2008–2014. The field protocol included (1) tagging the focal whale from a small rigid-hulled inflatable boat (RHIB) using a hand-held pole, (2) visual and VHF tracking of the tagged whale, and (3) recovery of the released tag (after 10–15 h of recording). On four occasions a second ‘non-focal’ whale was tagged (Table 1).

**Table 1:** Summary of analyzed data.

				Duration (h)		Dives ≥ 31 m	
Deployment Id	Body size	Calf	Experimental exposures	Total	Baseline	N	Duration (min)
gm08_150c	Small	1	MFAS, LFAS	4.8	0.9	0	
gm08_159a	Large	0	SIL, LFAS, MFAS, PB_KWF, PB_KWF	10.3	2.4	16	9.8
gm09_137b^1^	Medium	1		6.9	6.9	7	4.8
gm09_137c^2^	Small	0		7.6	7.6	5	4.5
gm09_138a^1^	Medium	0	LFAS, MFAS, SIL, LFASDS	9.9	3.2	11	9.2
gm09_138b^2^	Small	1	LFAS, MFAS, SIL, LFASDS, PB_KWF	17.2	3.2	31	7.5
gm09_156b	Large	0	SIL, LFAS, MFAS, LFASDS, PB_KWF	15.2	5.1	26	7.9
gm10_143a	Medium	1		8.8	8.8	11	5.7
gm10_152b	Small	0		1.6	1.6	2	2.5
gm10_157b	Medium	1	PB_BBN, PB_BBN	11.1	10.5	23	6.9
gm10_158d	Medium	0	PB_BBN, PB_KWF, PB_BBN, PB_KWF	7.4	2.9	0	
gm13_137a	Small	0	PB_KWM	6.3	2.8	14	4.0
gm13_149a	Large	0	PB_SON, PB_KWM	5.0	2.0	9	9.2
gm13_169a^1^	Large	1	PB_KWM, PB_SON, PB_BBN	6.6	2.1	11	4.7
gm13_169b^2^	Medium	0	PB_KWM, PB_SON, PB_BBN	6.7	2.1	6	2.1
gm14_180a^1^	Large	0	PB_SON, PB_KWM	8.0	2.4	0	
gm14_180b^2^	Medium	0	PB_SON, PB_KWM	8.3	2.4	0	
Sum				141.9	66.6	172	

*MFAS, 6–7 kHz sonar; LFAS, 1–2 kHz sonar; SIL, no-sonar control approach; PB_KWF/PB_KWM, playback of fish-eating/mammal-eating killer whale sounds; PB_BBN, playback of broad band noise (negative control). Superscripts following tag deployment numbers indicate pairs of whales (1 = focal, 2 = non-focal) that were tagged in the same aggregation of animals.*

Animal experiments were carried out under permits issued by the Norwegian Animal Research Authority (Permit No. 2004/20607 and S-2007/61201), in compliance with ethical use of animals in experimentation. The research protocol was approved by the University of St Andrews Animal Welfare and Ethics Committee and the Woods Hole Oceanographic Institutional Animal Care and Use Committee. The sound exposure experiments were designed and conducted within the 3S (Sea mammals, Sonar, Safety) research project (Miller et al., 2011).

### Experimental Exposures

The exposure experiments were designed and conducted within the 3S (Sea mammals, Sonar, Safety) research project. The full experimental protocol is described in Miller et al. (2011, 2012) and in Curé et al. (2012, 2013), and only briefly summarized here.

Tagged whales were exposed to blocks of transmissions (exposure sessions) of two or three of the following types of towed sonar: (1) Mid frequency active sonar (MFAS) 6–7 kHz hyperbolic upsweep, (2) Low frequency active sonar (LFAS) 1–2 kHz hyperbolic upsweep, or (3) LFAS 1–2 kHz hyperbolic downsweep. Sonar signals were 1 s in duration and were transmitted at 20 s intervals during exposure sessions. Each session transmitted one of the signal types for 25–80 min, with source levels increasing over the first 10 min of the exposure session (Appendix Table A1) following a mitigation protocol SAKAMATA (von Benda-Beckmann et al., 2012). The source (SOCRATES, TNO, the Netherlands) was towed from a source ship (55 m R/V H.U. Sverdrup II) toward the whale subject at a depth of about 55 m (range 35–100 m) and source levels (dB re 1 μPa m) ranged from 152 to 214 dB for LFAS and from 158 to 199 dB for MFAS. Moreover, whales were also subject to a no-sonar control, i.e., sonar source towed from approaching ship but without any transmissions, in order to separate potential effects of the approaching source from effects of sonar. The approach started at 6–8 km range to the tagged whale, and ended 5 min after the point of closest approach. The order of signal type was changed across tag deployments to enable evaluation of order effects, and all exposure and no-sonar control sessions had at least an hour between them (Miller et al., 2011, 2012).

In addition, sound playback experiments were conducted from a small motor boat (<10 m) that was stationed at ∼800 m range from the tagged whale at the start of each playback, and was allowed to drift over the course of the playback (Curé et al., 2013, Table 1). The stimuli included LFAS sounds (as above, but lower source levels), natural sequences of fish-feeding killer whale sounds (recorded locally in North Norway), mammal-feeding killer whale sounds (recorded in the Northeast Pacific), and broadband noise controls prepared from the non-calling periods of the killer whale recordings (amplified to achieve equal power to the killer whale sounds). All the playback stimuli transmitted from the small boat were 15 min (2008–2010) or 30 min (2013–2014) in duration and were broadcast at source levels of 145–151 dB re 1 μPa m, which is the typical source level of killer whale vocalizations (Miller, 2006).

For analysis, the received level of the towed sonar signals was estimated as the maximum sound pressure level over a 200 ms window (SPL_*max*_; dB re 1 μPa) (Miller et al., 2011). By design, the near-stationary playback sounds were received by the tag at relatively low and constant levels, and so their received level was not included in the analysis. Data were excluded from the beginning of the tag record until the end of tagging operations (when the boat used for pole-tagging was no longer active in the vicinity of the whale) (Isojunno and Miller, 2015). The data after tagging, but preceding any experimental control or sound exposure, was considered to be baseline data.

### Individual Data

Association with a calf was recorded during field observations when an adult-sized animal was tightly paired with a calf during the majority of its time at surface (over the entire duration of the tag deployment). Body size class was determined by combining field estimates (small/medium/large adult), and where available, estimates of dorsal fin size from good quality photographs of the tag attached to the dorsal fin of the whale (Isojunno et al., 2017). The base of the dorsal fin (Augusto et al., 2013) was measured in perpendicular photographs, and scaled to known length of the tag. The body size classes determined by field notes and photographs were compared to tag-derived fundamental fluke stroke frequency, a potential proxy for the body size of swimming animals. Sato et al. (2006) showed that fundamental stroke frequency correlates with the body mass of a wide range of breath-hold divers.

### Tag Data Processing

Depth, pitch and roll data (derived following Johnson and Tyack, 2003, decimated at 5 Hz) were assessed visually in a custom-built program in MATLAB 8.6 (MathWorks, Natick, MA, United States) to mark breath times in the time series (Appendix Figure A1). The method of detecting breaths from single-breath surfacings has been previously established in killer whales (Miller et al., 2010; Roos et al., 2016) and pilot whales (Wensveen et al., 2015). In most cases, breaths could also be heard in the acoustic record of the tag (Appendix Figure A2), but these were not included in the detection due to variable ambient noise conditions and gaps in the acoustic record. Near-surface behaviors with uncertainty about the number or timing of breaths were marked as “surface intervals.” These intervals could include logging behaviors where animals were near-stationary at the surface occasionally breathing (Miller et al., 2010; Isojunno et al., 2017). Movement data extracted from the DTAG were then summarized for each inter-breath-interval (IBI, between the end of the last breath/surface interval and the start of the next breath/surface interval). The time series were analyzed at 5 Hz sample rate, which defined the shortest breath duration as 0.2 s. Most analyses focused on the IBIs and did not attempt to estimate number of missed breaths during surface intervals, except for Model 3 (please see Section below for further details).

The fundamental stroke cycle frequency was extracted manually from periodograms (512-point spectral density, frequency resolution of 9.8 Hz) of high-pass filtered tri-axis acceleration and pitch (1st order Butterworth at 0.3 Hz) for each tag record (Sato et al., 2006). All three axes of the acceleration were plotted, but typically only one axis showed a clear peak indicating fluke strokes. As well as the peak, also lower and upper values were extracted by selecting frequencies that encompassed the whole width of the peak.

Time series of fluke strokes were generated using an automated detector based upon cyclic variation in pitch (Johnson and Tyack, 2003; Tyack et al., 2006). A first-order butterworth high-pass filter with a deployment-specific cut-off frequency (set to 0.8 times the tag’s fundamental stroke frequency) was used to smooth the pitch data prior to detection. We were able to assume minimal effects of tag placement because tags were always placed at or near the dorsal fin, and therefore set constant detection parameters for all tags. To assign the detection parameters, two example sets of 10 fluke strokes were detected manually for every tag. A fluke stroke was counted whenever there was a cyclic variation in the pitch deviation with peak-to-peak magnitude greater than a threshold of one degree (1°). The minimum and maximum allowable duration for the fluke strokes was set to 0.96 and 3.6 s, respectively.

We aimed to select a depth threshold for dives that would define a recovery period near the surface before resuming another deep dive. To achieve this, a range of depth thresholds (1–600 m) at 1 m resolution were used to calculate inter-dive intervals (IDI) between consecutive deep dives. To ensure sufficient data in each analyzed IDI, we selected a depth threshold that resulted in a minimum of 40 s IDI duration. The median IBI of the data was 20 s, and so this minimum IDI duration of 40 s represented a period of time that was likely to contain more than one breath.

**FIGURE 1:**
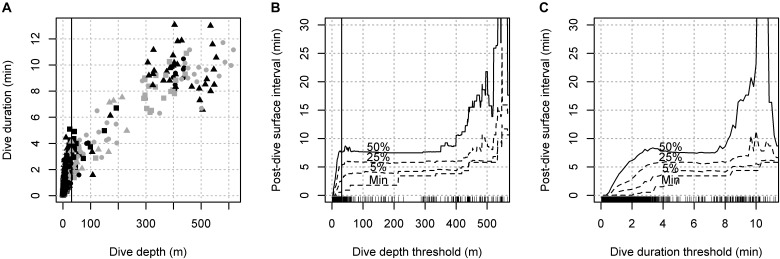
Selection of depth threshold for deep dives. **(A)** Relationship between the maximum dive depth and dive duration for all marked inter-breath intervals. Individuals associated with calves are shown in gray, while symbols indicate individual size category (triangle: large, circle: medium, small: square). **(B,C)** Minimum and percentiles (5, 25 and 50%) for post-dive surface interval durations between dives defined at different thresholds for dive depth and dive duration. Vertical lines in the left and middle panels show the selected threshold for deep dives (31 m).

There was a clear correlation between dive depth and duration, and both deeper and longer dives were followed by longer post-dive surface intervals (PDSIs) (Figure 1). The minimum PDSI duration increased gradually with increasing thresholds (∼1 min/100 m), while the median PDSI interval increased more rapidly initially as thresholds were increased for short or shallow dives, and became nearly constant for longer (>4 min) and deeper (>200 m dives). The depth threshold that resulted in a minimum of >40 s of PDSI duration was 31 m (Figure 1C), which was used to define dives for the rest of the analysis. We used a depth threshold rather than a duration threshold to avoid truncating the duration data of the resulting dives that were used to detect relationships between dive duration and subsequent breathing behavior.

### Statistical Analysis

To quantify breathing behavior related to diving, we define “net diving cost” (DC) as the number of additional breaths taken at surface due to a previous dive behavior, additional to a baseline level of breathing that is unrelated to diving (“non-recovery rate”, NRR). Included in the DC is a “locomotion cost” that we define as the number of breaths taken per fluke stroke. Locomotion costs are additional to a basal diving cost (δ) that is related to the duration of previous breath-holds alone.

We fitted four models: Model 1 was used to estimate whether longer dives would result in elevated breathing and an extended time to return to a baseline (non-recovery) rate, and whether breathing rates were also elevated prior to dives in an anticipatory response (Research question #1). Model 2 aimed to quantify how dive duration, locomotion effort and individual variables (body size, association with a calf) might influence the apparent net diving cost, i.e., the average number of breaths animals take to recover from a particular dive, additional to a breathing rate that is expected when animals have not been diving. This model addressed whether breathing behavior was linked to diving behavior (Research question #1), and our second research question about individual differences in post-dive recovery and breathing rate. However, because these models could only incorporate the effect of a single dive to a single pre- or post-dive surface interval (PDSI), they could not be used to predict breathing behavior outside of deep-diving periods, where multiple shallow dives could be conducted in quick succession. Therefore, Model 3 was developed to predict breathing behavior across all of the data, including the sonar exposures, as a function of all previous dive history. Model 4 was then used to test sound exposure effects (Research question #3), given the expected breathing rate under Model 3 (dive history + individual variables).

Generalized additive mixed models (GAMMs) were first used to model baseline breathing rate variability during PDSIs (Model 1: loading and recovery, Model 2: post-dive breaths) in order to incorporate individual as a random effect, account for serial correlation, and to test for non-linear relationships. A new cumulative model was then developed that assumed time-decaying additive effect of all previous dives on breathing rate (Model 3). The final Model 4 was also fitted as a GAMM to fully incorporate serial correlation while estimating the effects of sound exposure on breathing rate. In order to account for the baseline variability in this model, predicted breathing rate from the best cumulative model (Model 3) was included as an explanatory variable along with experimental exposure covariates (received level, type of exposure).

Experimental exposure data were excluded in Models 1–3. For Model 1 and 2, only data within 30 min preceding and/or following a dive were included. All data were included in the exposure effects Model 4. In all four models, IBIs (=submergence periods) shallower than the 31 m depth threshold were considered to be breathing behavior and were therefore included as response data. IBIs exceeding the depth threshold were considered to be dives and were therefore excluded as response data, but included as explanatory variables in the models. Please see Supplementary Material for the R scripts to fit, diagnose and interpret each model.

#### Model 1: Loading and Recovery

Inter-breath interval (IBI, s) was used as the analysis time unit to estimate how breathing patterns might change prior to and following dives. To focus the analysis on breathing rate, the inverse of the IBI (1/IBI = breathing rate, calculated over a single IBI) was used as the response variable, i.e., the IBI was used as the time unit for breathing rate to define an instantaneous breathing rate (IBR, min^−1^). For example, an IBI of 15 s equals to an IBR of 4 breaths per minute. Using IBR instead of counting breaths over fixed time periods avoided discretization of the data and the need to analyze periods where the exact number of breaths was not known (i.e., ‘surface intervals’ define above).

IBR was modeled as a Gamma distributed response variable in a GAMM (package mgcv in r) with log link function. The model was specified 1st order auto-regressive serial correlation within each inter-dive-interval, which was included in the model as a nested random effect along with the deployment number. Model residuals were checked for serial correlation and distributional assumptions (acf and gam.check functions). Time to next deep dive, time since previous deep dive and deep dive duration were included as smooth covariates (thin plate splines). To allow for different duration and shape of response as a function of previous deep dive duration, time since previous deep dive and dive duration were included as a non-linear interaction term (tensor product).

#### Model 2: Number of Post-dive Breaths

The analysis of post-dive number of breaths aimed to quantify how many additional post-dive breaths were taken as the dive duration (apnea) and stroking effort (proxy for locomotion costs) increased. The total number of detected breaths was calculated within post-dive surface intervals (PDSI) that lasted up until the next dive, or until a maximum set time window duration (*t_win_*) was reached. The *t_win_* duration was selected based upon the results of Model 1 (loading and recovery), but we also conducted sensitivity analyses on its selection. The post-dive number of breaths was modeled as a Poisson response variable, again in a GAMM that incorporated the deployment as a random effect, and allowed for serial correlation between subsequent dives (1^st^ order auto-regressive correlation). The model was specified an identity link, and the dispersion parameter for the Poisson family was estimated rather than fixed (option ‘quasipoisson’).

The duration of the PDSI (always less than or equal to the maximum duration *t_win_*) was included as an explanatory variable (*PDSI.dur*) in the model in order to account for any variation in respiration rates that was unrelated to the previous dive. Individual variation in this rate was incorporated in the random effects of the model, but we also tested whether calf association or body size class could influence this rate. This was achieved by specifying interactions between *PDSI.dur* and individual class as candidate covariates. Individual body size classes and calf association were coded as presence-absence variables in the model. The intercept referred to individuals without a calf (*calf* = 0) in the medium body size category (*small* = 0, *large* = 0).

In order to account for uncertainty related to any missed number of breaths, a metric of missed breaths was calculated for each PDSI and included as an explanatory variable (*missed.num*). Missed number of breaths was considered for those surface intervals where individual breaths could not be marked (Appendix Figure A1) and that were long enough in duration (≥5 s, selected based on audited inter-breath intervals; Appendix Figure A2) to potentially contain more than a single breath. The expected number of missed breaths was calculated based on the surface interval duration and an expected IBI, calculated as a median over the two IBIs immediately prior and one IBI following the interval.

AIC model selection was used to determine which variables best explained the post-dive number of breaths. All models included *PDSI.dur* and *missed.num* as explanatory variables, and up to six candidate covariates in a global selection (i.e., testing for all covariate combinations). Candidate covariates included dive duration (min), number of fluke strokes, fluke stroke rate, as well as interactions between dive duration and the individual covariates (presence/absence of calf, and body size) that tested for the effect of calf association and body size on the number of breaths taken per minute of diving. In order to estimate net diving costs per minute, which includes cost of locomotion effort, and also fit models where locomotion effort was estimated separately from basal diving costs, the AIC model selection was conducted both with and without stroking effort. AIC difference of 2 units was considered to be sufficient to support more complex models. We also tested whether each term explained significant variation in the response data (Wald tests using the Bayesian covariance matrix for the coefficients).

#### Model 3: Cumulative Model

The cumulative model quantified dive recovery as an elevated post-dive breathing rate that exponentially decreases to a level *β*_i_ that is no longer influenced by previous diving history. This level, or “NRR”, was allowed to vary between individual size classes and between individuals with and without a calf in a linear regression (Appendix B). Net diving costs *θ*_k_ were specified as an additive effect of dive duration (basal diving cost (*δ*), with the coefficient *δ*_i_ varying between individual size classes and calf association) and number of fluke strokes (an individual-average parameter). Breathing rate was then modeled as the sum of the baseline rate *β*_i_ and the exponentially decaying net diving costs of all previous dives. The exponential decay was expressed in terms of the number of breaths achieved during the mean lifetime *τ*_k_ of the decay, i.e., the time at which breathing rate was expected to reduce to 1/e (∼37%) of the initial breathing rate *ρ*_k_. The initial breathing rate was assumed to increase linearly with the net diving cost *θ*_k_, up to a maximum (*ρ*_max_). The model assumed the resulting breathing rate to be a Gamma-distributed variable (DeRuiter et al., 2013). Please see Appendix B and Supplementary R code for further details on the model formulation and fitting.

#### Model 4: Exposure Effects

Similar to the loading and recovery model (Model 1), IBR and stroke rate were modeled as a Gamma distributed response variable in a GAMM specified with first-order autoregressive serial correlation and identity link function. However, the model could now be fitted to all of the shallow (<31 m) inter-breath intervals, and not restricted to post dive surface intervals of ≥31 m dives alone. Exposure covariates were included in the model to allow breathing rate and stroke rate to decrease or increase during the experimental exposures. Exposure covariates included the received level of the sonar approach (SPL_max_), calculated as the maximum received SPL in a 5 min window prior to the IBI start time, and six presence/absence covariates which were set to one (1) during and 5 min post of the exposure session, and zero (0) otherwise. The presence/absence exposures included the no-sonar approach (NS), broadband noise control playback (PB_BBN), playback of sonar sounds (PB_SON), and playback of fish-eating and mammal-eating killer whale sounds (PB_KWF and PB_KWM, respectively). We also included an order effect, which was set to absent (0) during the first sonar exposure, and present (1) for the subsequent sonar exposures. In order to account for the effects of dive history and individual class, Model 4 included predicted IBR (pred.IBR) from the cumulative Model 3 as a covariate. No other covariates than the exposure covariates were included in Model 4 because the pred.IBR included expected variation from all the baseline covariates included in Model 3 (dive duration, number of fluke strokes, body size class and calf association).

AIC model selection was not possible due to the autocorrelation structure and non-normal errors of the Gamma model. Instead, Wald tests were conducted on each covariate fitted in a full model, and a reduced model was fitted with those covariates that tested significant at 5% level. The full model included pred.IBR, all the six presence/absence exposure covariates and two smooth covariates: SPL_max_ specific to the 6-7 kHz exposure (MFAS-SPL_max_), and SPL_max_ specific to the 1–2 kHz LFAS exposure (LFAS-SPL_max_). The smooth covariates were selected using the shrinkage penalty terms on the thin plate smooth (Marra and Wood, 2011).

## Results

### Data

A total of 141.9 h of tag data from 17 whales were analyzed, of which 66.6 h were baseline data (Table 1). 13 out of 17 tagged whales were exposed to naval sonar and/or sound playbacks and the other 4 tag deployments only contained baseline data. The median IBI (mean of individual medians) was 20 s (3 breaths min^−1^) and the median surface interval 0.4 s (note 5 Hz resolution). Across the 14 animals for which photographs were available, there was a reasonable concordance between the field estimated body size class (small, medium, and large) and the size of the dorsal fin (Appendix Figure A3). As predicted, the fundamental fluke stroke frequencies were highest for individuals in the smallest body size category (0.58–0.72 Hz) and lowest for individuals in the largest body size category (0.45–0.55 Hz); however, there was considerable overlap with the frequencies for the medium category (0.5–0.7) (Appendix Figure A3).

The maximum dive duration (13.9 min) and maximum dive depth (617 m) were achieved by individuals with large and medium body size, respectively. Individuals with small body size conducted shorter and shallower dives (max 10.9 min and 446 m, *N* = 4). The average dive duration and depth for individuals with large body size were 7.8 min and 323 m (*N* = 4), respectively, compared to 4.6 min and 139 m (*N* = 4) for individuals with small body size (Table 1 and Figure 1A).

### Model 1: Loading and Recovery

**FIGURE 2:**
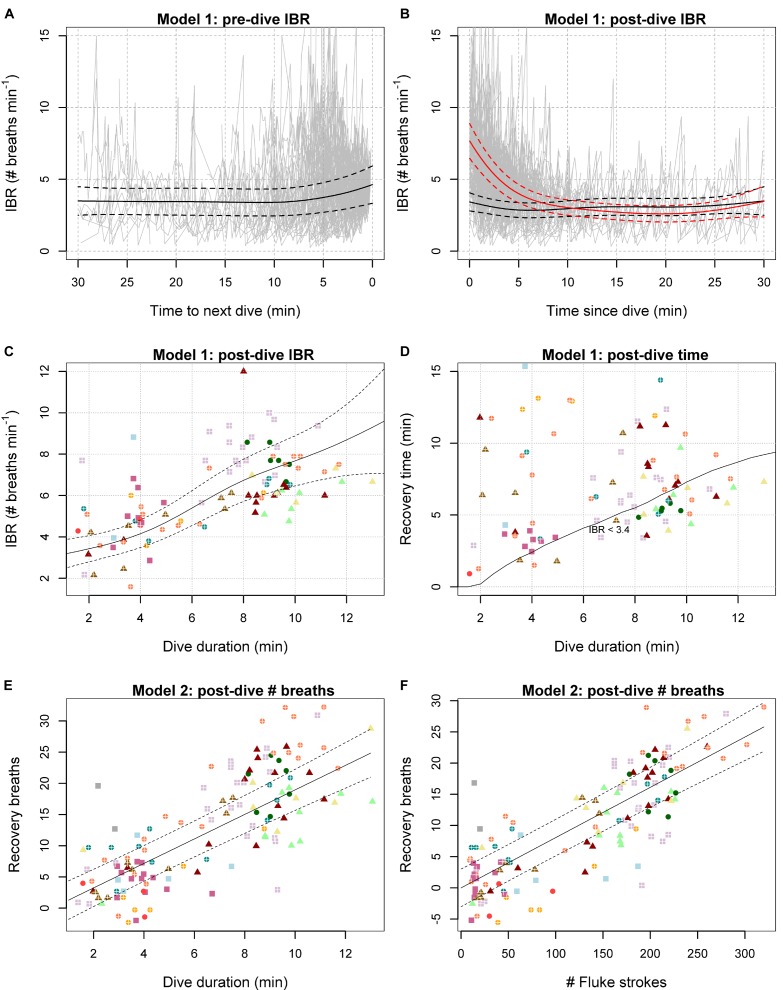
Model 1 and 2 estimates overlaid with observed data. **(A,B)** Model estimated IBR preceding **(A)** and following **(B)** dives (≥31 m), overlaid with observed IBR (gray). Black lines show estimates for a 2-min dive, and red lines show estimates for a 10-min dive. **(C–F)** Model predictions are shown as solid lines and ±2SE as dashed lines. Symbols indicate individual type (square: small body size, triangle: large body size, crosses: calf association), while colors indicate different individuals. **(C)** Observed (symbols) vs. predicted post-dive initial IBR (with 95% CI) as a function of dive duration. **(D)** Time for recovery of post-dive breathing rate to baseline levels as a function of dive duration and model predicted time it would take for the IBR to decrease to threshold levels (IBR < 3.4). Symbols show observed post-dive surface interval duration. **(E,F)** Model predictions of recovery breaths as a function dive duration (Model 2b) and number of fluke strokes (Model 2a). Symbols give observed values minus predicted based upon number of missed breaths and post-dive surface interval duration (i.e., ‘recovery breaths’ excluding a baseline level that is not related to diving).

The IBR was elevated both immediately prior to, and immediately following dives to ≥31 m depth (Figures 2A,B). The model including the non-linear interactions allowed the IBR recovery to vary with previous dive duration (Figure 2B). Initial IBR was estimated to increase linearly following longer duration dives (Figure 2C), and subsequently take longer to recover to an intercept level (Figure 2D and Appendix Figure A5). Fitting time since dive and dive duration as smooth main effects instead reduced the model fit (adjusted *R*^2^ decreased from 0.403 to 0.372), although the main effect was still significant (*p* < 0.001). In contrast, duration of the subsequent dive was not supported as a main effect (*p* = 0.169), and including it as a non-linear interaction with time to next dive did not improve the model fit (*R*^2^ = 0.403) either. From this model, estimated IBR immediately prior to a 2-min vs. 10 min dive were not significantly different (5.4, *SE* = 1.2 vs. 4.7, *SE* = 1.0 breaths min^−1^ respectively), indicating that pre-dive IBR did not vary with subsequent dive duration. Therefore, the best model that was used to make inferences about pre- and post-dive IBR (Figures 2A–D) excluded duration of the next dive. No serial correlation remained in the model residuals (Appendix Figure A4).

The best model estimated the average recovered IBR to be 3.4 breaths min^−1^ (SE 0.5) (30 min prior to, and 30 min following a ≥ 31 m depth dive). IBR was predicted to be elevated and more variable immediately before dives that were 2 min or longer in duration (4.6 breaths min^−1^, *SE* = 0.65) (Figure 2A). IBR was less variable, but on average close to the recovered level immediately after a 2-min dive (3.4 breaths min^−1^, *SE* = 0.3) while the IBR was predicted to more than double immediately following a 10-min dive (7.7 breaths min^−1^, *SE* = 0.6) (Figure 2B). The initial IBR was estimated to increase by 0.52 breaths min^−1^ for every 1 min increase in dive duration (Figure 2C). The model predicted the IBR to return to the baseline (3.4 breaths min^−1^) after 0.2, 7.3, and 9.5 min since 2-, 10-, and 14-min dives, respectively. For ≥31 m dives lasting 2 min or longer, the recovery time increased by 0.8 min for every 1 min increase in dive duration (Figure 2D and Appendix Figure A5).

### Model 2: Number of Post-dive Breaths

The duration of the time window over which breaths were calculated (*t*_win_) was set at 10 min. The selection was based upon the Model 1 estimated recovery time (9.5 min) following the maximum dive duration observed in the data (13.8 min) (Figure 2D). Sensitivity analysis indicated that parameter estimates were relatively stable for *t*_win_ duration longer than 8 min, except the coefficient estimate for missed number of breaths. The effect of missed breaths gradually decreased as the *t*_win_ duration increased (Appendix Figure A6).

AIC model selection supported the inclusion of number of fluke strokes (df = 1, *F* = 231, *p* < 0.001), but not fluke stroke rate, dive duration or effect of individual type. The model (AIC = 789) explained 74% (adj. *R*^2^) of the response data. The scale parameter of the model was estimated as 0.77. AIC increased when dive duration was included in the model, either as an interaction term with number of fluke strokes (ΔAIC = 0.7) or as a main effect (ΔAIC = 1.8). Including stroke rate, or including both stroke rate and dive duration changed the AIC only very slightly (−0.1 and 1.2, respectively). A model with dive duration and stroke rate instead of stroke number increased the AIC by 16 units (AIC = 805, adj. *R*^2^ = 0.70). In this model, both dive duration and stroke rate were supported (df = 1, *F* = 80, *p* < 0.001, and df = 1, *F* = 16, *p* < 0.001, respectively). However, number of fluke strokes and dive duration were strongly correlated (Spearman’s rho = 0.86, *n* = 133 non-exposed dives). Excluding both metrics of stroking effort from the model selection (to estimate net diving costs per minute of diving; see Methods), the best AIC model (the simplest within models < 2 AIC units; AIC = 817, *R*^2^ = 0.68) retained dive duration as a main effect (df = 1, *F* = 158, *p* < 0.001) and no individual covariates. The lowest AIC model included the interaction between dive duration and calf but the AIC of this model was only slightly lower (0.3 units).

In the best model excluding stroking effort, the total number of breaths taken during the post dive surface interval (PDSI) was estimated to be 2.7 breaths at the start of the PDSI (*SE* = 1.5), plus 1.9 (*SE* = 0.1) additional breaths per minute of PDSI, and minus 0.2 (*SE* = 0.1) breaths per each calculated missed breath (Table 2). Net diving costs were interpreted to be any additional recovery breaths taken above this baseline level (Figures 2E,F). When stroking effort was not considered, diving to ≥31 m was estimated to cost 1.7 breaths (*SE* = 0.2) per minute of diving. In the best AIC model including number of strokes, rather than dive duration, the apparent cost of each fluke stroke was estimated to be 0.081 (*SE* = 0.005) breaths (Figure 2F).

**Table 2:** Parameter estimates From Model 2a (best model for the number of post-dive breaths) and 2b (best model excluding stroking effort).

Model	Covariate	Estimate	*SE*	*t*	*p*-value
2a	Intercept	2.677	1.509	1.8	0.119
	No. of missed breaths	−0.195	0.062	−3.2	0.004
	PDSI duration (min)	1.899	0.120	15.7	<0.001
	No. of fluke strokes	0.081	0.005	15.2	<0.001
2b	Intercept	−0.409	1.570	−0.4	0.794
	No. of missed breaths	−0.182	0.069	−2.5	<0.010
	PDSI duration (min)	1.895	0.132	14.2	< 0.001
	Dive duration (min)	1.674	0.207	12.6	<0.001

*t-test statistic and p-values are shown for the null hypothesis that the estimate is zero. PDSI: post-dive surface interval.*

### Model 3: Cumulative Model

The best AIC model (lowest AIC and also the simplest < 2 AIC units) included the effect of small body size and calf association on the basal diving cost (δ), and excluded the effect of large body size class (Table 3). Including small body size and calf association in the model improved the model without any individual effects by 20 and 4 AIC units, respectively. There was clear evidence that the small body size effect was different from zero (*z* = 9.9, *p* < 0.001) while the calf effect was not significantly different from zero (*z* = −0.2, *p* = 0.8).

**Table 3:** Cumulative model selection.

ID	# par.	AIC	ΔAIC	*R*^2^	ε	Calf	Small	Large
6	11	37269.4	0.0	32.3	14.2	−	+	
1	12	37281.2	11.8	32.1	14.3	−	+	+
3	10	37300.2	30.8	33.0	14.3	−		
7	11	37300.6	31.2	33.0	14.3	−		−
8	11	37313.8	44.4	33.8	14.4		+	+
4	10	37315.9	46.5	33.8	14.4		+	
2	9	37320.1	50.7	33.3	14.4			
5	10	37320.4	51.0	33.2	14.4			+

*The direction of individual type effects on diving costs are indicated as ± when they were included in the model.*

The best cumulative model explained 32% (*R*^2^) of the variation in breathing rate (inter-breath intervals of dives < 31 m). The model did not predict IBR values higher than 8.6 breaths min^−1^, resulting in some positive skew in the residuals (Appendix Figures A7A–C). However, the residuals of the model indicated good fit immediately after long (>2 min) dives (Appendix Figure A7D). Please see Appendix C for the full time series of model predictions.

The best cumulative model estimated each fluke stroke to cost 0.086 breaths (95% CI [0.082, 0.089]), additional to the basal cost of breath-hold diving per minute of dive time (basal diving cost δ). Individuals with small body size were estimated to have the greatest basal costs (0.99 breaths min^−1^ diving [0.67, 1.45]), but were also estimated to recover to a lower level of breathing rate (non-recovery rate NRR, 2.66 breaths min^−1^ [2.49, 2.83]) than individuals with medium or large body size class (Table 4). Conversely, individuals in the medium or large body size class had the lowest basal costs (0.30 [0.26, 0.35] breaths min^−1^ diving) and the highest NRR (2.89 breaths min^−1^ [2.82, 2.95]). Besides the non-significant negative effect on basal costs of diving, association with a calf was estimated to significantly increase NRR by a further 6% [3–9%] in each body size class (Table 4).

**Table 4:** Parameter estimates from the best cumulative model.

Parameter	Interpretation	Estimate (95% CI)	Z	p-value
ε	Observation error	14.24 (13.06, 15.5)	60.4	<0.001
β_0_	Intercept for NRR	1.06 (1.04, 1.08)	100.8	<0.001
β_1_	Calf effect on NRR	0.06 (0.03, 0.09)	4.4	<0.001
β_2_	Small body size effect on NRR	−0.09 (−0.13, −0.04)	−3.8	<0.001
β_3_	Large body size effect on NRR	−0.07 (−0.09, −0.04)	−6.0	<0.001
δ_0_	Intercept for DC	−1.20 (−1.35, −1.05)	−15.6	<0.001
δ_1_	Calf association effect on DC	−4.61 (−43.50, 34.27)	−0.2	0.816
δ_2_	Small body size effect on DC	1.19 (0.95, 1.42)	9.9	<0.001
φ	Cost of locomotion per stroke	−2.46 (−2.50, −2.42)	−123.5	<0.001
r	Increase in initial BR per DC	0.25 (0.24, 0.27)	−62.0	<0.001
p_max_	Maximum initial BR	5.44 (5.17, 5.72)	65.3	<0.001

*Regression coefficients (β_0_, β_2_, β_2_, β_3_, δ_0_, δ_2_, δ_3_) are given at link (log) scale. BR, breathing rate; NRR, non-recovery rate; DC, basal diving cost.*

The cumulative model was used to estimate the net diving cost for each observed dive (total breaths per ≥31 m dive). All dives to >300 m depth cost more than 11 breaths. The maximum estimated per-dive cost across all dives, 27.5 breaths, was achieved by a medium individual with a calf (gm10_157b) (Appendix Figure A8A). For individuals without calves, the average per-dive cost increased with body size (individual-average net per-dive cost 7.8, 12.5, and 17.0 breaths per dive, respectively).

### Model 4: Exposure Effects

The effects that tested significant at 5% level in the full model were pred.IBR (df = 1, *F* = 1028, *p* < 0.001), PB_KWM (df = 1, *F* = 7.3, *p* = 0.007), LFAS-SPL_max_ (df = 1, *F* = 20, *p* < 0.001) and the order effect SON_2 (df = 1, *F* = 7.2, *p* = 0.002). There was little support for any of the other covariates (*p* > 0.18). LFAS_SPL_max_ was also supported when the full model included SPL_max_ (combining both received levels of both LFAS and MFAS) instead of MFAS-SPL_max_, indicating a signal-specific effect. However, LFAS transmitted from the near-stationary playback system (PB_SON) was not supported in the full model (df = 1, *F* = 0.4, *p* = 0.52). Wald test results were almost identical for each effect in the final reduced model, with the least significant effects (the order effect and PB_KWM) still gaining support at *p* = 0.01. This was reassuring as the model residuals retained some serial correlation even with the fitted autocorrelation structure (Appendix Figure A9).

**FIGURE 3:**
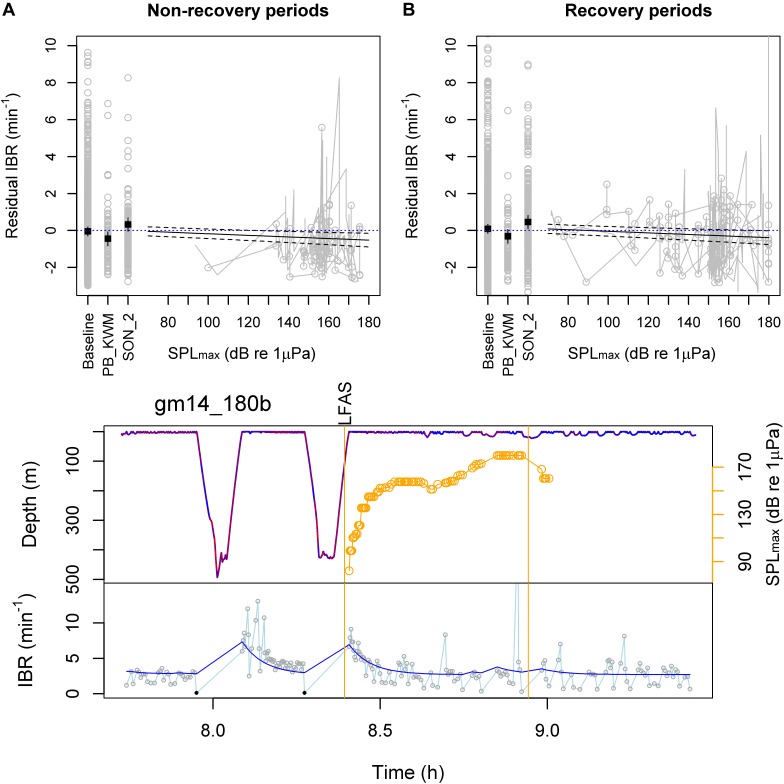
Model 4 estimated effects of experimental exposures and example time series. **(Top)** Observed (gray) and Model 4 predicted (black) residual instantaneous breathing rate (IBR; above rate expected by Model 3) are shown as a function of received level (SPL_max_) during the first sonar approach, during subsequent sonar approaches (SON_2) and during mammal-feeding killer whale playbacks (PB_KWM). Non-recovery **(A)** vs. recovery periods **(B)** were defined as below or above 110% of the non-recovery baseline rate estimated by the cumulative model for each individual type. Horizontal dotted lines show zero residual IBR as a reference. **(Bottom)** Example time series of IBR (connected gray circles) overlaid with the cumulative model estimates (dark blue line). The whale’s dive profile is color-coded by fluke stroke rate (red: higher rate) and overlaid with 1–2 kHz LFAS sonar exposure start and end time (vertical lines) and received SPL_max_ (maximum calculated in a 5 min time window prior to each breath; hence also 5 min past exposure end time). IBR of ≥31 m dives were excluded as response data from the model, and are shown as black solid circles.

The final model explained 32% of the response data during pre-exposure baseline, 43% during exposures and 27% during post-exposures (*R*^2^) (*N* = 13 subject individuals, Table 1). The model estimated IBR to decrease from 3.1 breaths min^−1^ (*SE* = 0.2) during baseline to 2.7 breaths min^−1^ (*SE* = 0.2) during the mammal-feeding killer whale playbacks, and to 2.6 breaths min^−1^ (*SE* = 0.2) when the maximum LFAS-SPL_max_ reached 180 dB re 1 μPa during the first sonar exposure. Conversely, the duration of shallow < 31 m dives increased (Figure 3C). The IBR was estimated to increase during the subsequent sonar exposures (3.5 breaths min^−1^, *SE* = 0.2). Reduced breathing rates were evident both inside and outside expected dive recovery periods (Figure 3). Figure 3 shows breathing rate above expected breathing rate from Model 3. For raw observed values and predictions, please see Appendix Figure A10.

## Discussion

Our results demonstrate that the number, frequency, and timing of breaths of long-finned pilot whales can be linked to diving history. Our data showed a marked and consistent increase in IBI immediately following dives ≥ 31 m depth, and subsequent recovery that was dependent on the duration and locomotion effort of the dive (Figure 2). These data support the hypothesis that breathing patterns may be particularly stereotyped following maximal performance. Apparent basal diving costs (breaths min^−1^ diving; excluding locomotory costs, but additional to a baseline level of breathing unrelated to diving) were the highest for smaller individuals (Table 3), as expected due to their higher mass-specific metabolic rate. Apparent costs were also estimated per unit of locomotion effort (cost per stroke; Williams et al., 2004; additional to a basal diving cost related to the duration of breath-hold alone), though since oxygen extraction rate and tidal volume might vary, we still do not know how much oxygen was exchanged per breath to assess specific metabolic costs. A new cumulative model was developed to formalize the exponential recovery from dives and accumulate the effects of multiple previous dives, which showed good fit to the data. We were able to assess effects of sound exposures on breathing patterns as additive to those expected by changes in diving behavior and individual variation by including the expected breathing rate from the cumulative model as an explanatory variable.

### Using Breathing Patterns as an Indicator for Diving and Locomotion Costs

As expected, both breath-hold duration and stroking effort increased the number of breaths taken per dive, with an increase in both the initial IBR, and the duration over which the IBR was estimated to remain above a recovered level (Figure 2). Including total stroking effort removed the effect of dive duration in the model for post-dive number of breaths (Model 2), revealing that stroking effort explained more variation in breathing behavior than breath-hold-duration. Of course, the two are correlated, and so the total number of fluke strokes in a dive could inform both about locomotion effort and dive duration (Halsey, 2017). Stroking effort may have also captured some variation due to body size, because smaller animals have a higher fluke stroke cycle frequency (results herein, Sato et al., 2006). Such exercise-modulated breathing behavior in long-finned pilot whales indicates that locomotion incurs a substantial energetic cost to their foraging. Such a pattern may be part of a “spend more, gain more” strategy for which high quality prey justify high energetic costs during foraging dives (Aguilar Soto et al., 2008). This interpretation is consistent with the muscle morphology of pilot whales (Velten et al., 2013), and fits within a general pattern in cetaceans where the predators’ maintenance and locomotion costs are determined by the quality of their prey (Spitz et al., 2012).

Breathing behavior can be expected to be more optimized immediately before or after dives that are more energetically and physiologically demanding, due to physiological requirement to re-establish homeostasis, as well as during periods of foraging when individuals maximize the proportion of time spent feeding at depth. As expected, dive behavior and subsequent breathing behavior were the most correlated after longer dives, and immediately after the dives (Appendix Figure A5). These breathing patterns may therefore represent an optimal strategy to maximize gas exchange, given a set of physiological constraints for loading O_2_ and eliminating CO_2_. For O_2_ loading, a key factor is the partial pressure of O_2_ (PO_2_) gradient between the air in the alveoli and the blood in the pulmonary arteries supplying the lung (e.g., Boutilier et al., 2001; Fahlman et al., 2009; Noren et al., 2012; Roos et al., 2016). After a long dive PO_2_ in the blood might be low (Meir et al., 2009; Noren et al., 2012) and this increases oxygen extraction rate per breath, compared to short dives. Diminished returns for increasing oxygen stores (i.e., surface time) has been the basis of marginal models for optimal diving (Kramer, 1988). However, eliminating CO_2_ from tissues and blood is expected to be slower than O_2_ uptake, and may therefore be more important in setting surface time (Boutilier et al., 2001). It is interesting to speculate whether the gradual increase in post-dive IBI (and conversely, exponential decay in post-dive IBR) may be an optimal strategy to eliminate CO_2_. Breath-by-breath measurements of end-tidal O_2_ and CO_2_ combined with an analysis of breathing patterns would help to elucidate how the timing of post-dive breath might track loading of O_2_ and/or eliminating of CO_2_. Compartment gas dynamic models (e.g., Fahlman et al., 2009; Kvadsheim et al., 2012) could also be used to test this hypothesis.

Our data also showed that breathing rates were elevated during deep diving (foraging) periods and immediately prior to dives ≥ 31 m depth. Recovered breathing rate was 3.4 breaths min^−1^ during post-dive surface intervals (Model 1), compared to 2–3 breaths min^−1^ outside the foraging periods (Model 3). During the foraging periods, there was a further anticipatory increase in IBR 5 min prior to a ≥31 m dive. Unlike post-dive IBR however, the increase was not dependent on the dive duration (Figures 2A,B). This result shows that individuals may load O_2_ (and/or remove CO_2_) to a fixed optimum level before a dive, indicating that the cost of anticipatory ventilation is small compared to the benefit of having flexibility in dive duration. Terminating dives early in response to an assessment of prey quality at depth can improve the overall prey encounter rate of a breath-hold diver (Thompson and Fedak, 2001). Long-finned pilot whales, similar to other odontocetes, can make such an assessment using echolocation, within the range of their biosonar. This interpretation is supported by the diving behavior of long-finned pilot whales in our study area, where demersal foraging dives (>300 m) are often preceded or followed by shallower echolocating dives, indicating exploratory behavior (Isojunno et al., 2017).

### Individual Differences in Apparent Diving Costs

Individuals in the smallest body size class incurred the highest basal diving costs (breaths min^−1^ diving) but maintained a relatively low breathing rate outside recovery periods (Table 3). As well as high mass-specific metabolic rate, small animals also have a smaller total capacity to store O_2_/CO_2_ in the body, which may further limit their aerobic dive duration. Medium and large animals conducted both longer and deeper dives than animals in the small body size class, suggesting that diving capacity was a limiting factor for small animals’ diving behavior. This greater diving effort resulted in greater total number of breaths taken per dive by larger bodied individuals not associated with a calf. While the effect of body size class was supported in the cumulative model, it was not supported in Model 2 for post-dive number of breaths. This was likely due to the cumulative model including the effect of the shallower dives, which constituted a larger portion of the dive data in small animals.

We also expected smaller animals to have higher mass-specific cost of locomotion for a given speed (Baudinette, 1991; Williams, 1999). In aquatic animals, the power that is needed to swim against hydrodynamic drag can be expected to scale with the product of swim speed^3^ and surface area of the body, which in turn scales allometrically with body mass (Bose et al., 1990). Due to the relatively small sample size, we were not able to include size-specific locomotion costs in our analyses. However, applied to larger datasets in the future, such a size-specific parameter or an allometric relationship could be incorporated in the cumulative model to more fully quantify the effects of body size on breathing behavior.

### Effects of Sound Exposures on Breathing Behavior

Energy mobilization is a significant component of the physiological stress response and can lead to an elevation of metabolic rate (Sapolsky et al., 2000). However, in marine mammals adapted to conserving oxygen, metabolic rates may be suppressed in response to stressful events such as forced submersion (Ponganis, 2015). Long-finned pilot whales may not be among the most behaviorally responsive cetacean species to sonar (Miller et al., 2012; Antunes et al., 2014; Harris et al., 2015) but a wide range of responses, including avoidance, cessation of foraging, and changes in vocal and social behavior have been observed (Rendell and Gordon, 1999; Miller et al., 2012; Sivle et al., 2012; Antunes et al., 2014; Wensveen et al., 2015; Visser et al., 2016). Long-finned pilot whales have also been shown to be attracted to playbacks of killer whale (a potential predator/food competitor) sounds (Curé et al., 2012 whose data is also included here, Bowers et al., 2018). If these behavioral responses were associated with a stress response, we expected a change in breathing behavior that would not be explained by recovery from previous breath-hold duration or stroking effort. Our results support this hypothesis, with Model 4 indicating a reduced breathing rate relative to diving behavior during playbacks of mammal-feeding killer whale sounds and at higher received sound pressure levels of sonar (1–2 kHz LFAS signals), but only during the first sonar exposures. For the subsequent exposures, there was an increase in breathing rate. This order effect is consistent with previous analysis of these data indicating that pilot whales change response tactic to repeated sonar exposures, with more variable responses during subsequent exposures (Isojunno et al., 2017). Previous analyses of the same dataset also found a greater effect of the LFAS signal compared to the MFAS (Sivle et al., 2012; Wensveen et al., 2015; Isojunno et al., 2017), similar to sperm whales (Curé et al., 2016; Isojunno et al., 2016).

The order effect and the similar reduction in breathing rate (13 vs. 16%) during the killer whale sound playbacks and the towed-source LFAS exposure suggests that perceptual effects (i.e., due to perceived risk/cost/opportunity), rather than sound exposure level alone, was driving the response. Indeed, the reduction during the first LFAS exposure (from 3.1 breaths min^−1^ down to 2.6 breaths min^−1^) was more than expected if the tagged whale matched its surfacings with the timing of the sonar pings every 20 s, i.e., 3 breaths min^−1^ as a means to reduce their acoustic exposure (Wensveen et al., 2015). Furthermore, a lack of similar response during the fish-feeding killer whale playbacks may indicate that long-finned pilot whales perceive the fish-eating and mammal-eating killer whales sounds as different levels and/or types of disturbance stimuli. While there was a reduction in breathing rate during the near-stationary LFAS playbacks as well, it was not statistically significant. This could have been due to the small number of sound exposures (*N* = 2, Table 1).

In terms of the energetic costs, the breathing rate lower than would be expected for the same diving behavior during the mammal-feeding killer whale playbacks and first sonar exposures may reflect (1) no or little change in metabolic rate due to increased tidal volumes, matching the energy requirements of a shallow diving response to a perceived risk, and/or (2) a suppressed metabolic rate and subsequent reduced need to take breaths, which may be part of a hypometabolic stress response. It is not possible to distinguish between the two possible explanations based on the breathing pattern alone. However, given the reduced breathing rate, it seems unlikely that the whales significantly increased their oxygen consumption beyond the energy required for repeated shallow diving. Our data also indicate that the reduced breathing is incompatible with long and active foraging dives, given the elevated breathing rates prior to ≥ 31 m dives and throughout deep diving periods. Such a cessation of foraging would imply an energetic cost in terms of lost feeding opportunities. Interestingly, breathing rates were also reduced by individuals that were expected to be recovering from deep dives and maintain high breathing rates during the exposures (Figure 3B), suggesting that the recovery breathing pattern could have been disrupted. It is possible that the behavioral response would therefore lead to additional physiological costs, especially if coupled with a hypometabolic response. However, using the same dataset Kvadsheim et al. (2012) found that the change in dive behavior associated with the response to the sonar did not lead to any increased risk of decompression sickness (nitrogen embolism) in pilot whales. The response could also indicate flexibility in the recovery breathing pattern, which after a cessation of foraging state no longer reflects an optimal breathing pattern to maximize dive time. Such flexibility could enable long-finned pilot whales to avoid visual detection by predators by staying submerged for longer periods of time in a shallow-diving behavior response.

### Conversion of Breathing Patterns to Estimates of Field Metabolic Rate

Our results indicate that long-finned pilot whale respiratory rates during resting and post-dive recovery are similar to other similarly sized marine mammals and cetaceans. An allometric equation between body mass and respiratory frequency for semi- and fully aquatic mammals predicted a resting respiratory frequency (Mortola and Limoges, 2006) for 800 kg mammal to be 2 breaths min^−1^ (500 vs. 1500 kg: 2.4–1.8 breaths min^−1^). In our data, the expected IBR varied between 2.6 and 8.6 breaths min^−1^ (Model 3), with breathing rates of 4–6 breaths min^−1^ sustained up to 5 min. The average deployment included 43% of time with IBR below 2 breaths min^−1^ at shallow depths < 31 m (range 18–62%), consistent with other studies showing that pilot whales spend large proportion of time near surface or shallow diving (Baird et al., 2002; Quick et al., 2017). These resting rates and a three- or four-fold increase during post-dive recovery are similar to what was reported for a 645 and 907 kg trained beluga whales: 1.6 during rest, 5.5 during swimming exercise and up to 9.6 breaths min^−1^ during post-dive recovery following dives to <300 m depths (Shaffer et al., 1997). The post-dive recovery time and number of required breaths had a remarkably linear relationship with dive duration, which might indicate that the whales did not reach a physiological constraint line (Horning, 2012) and were mostly diving within their aerobic dive limit (ADL) and without lactate accumulation in circulation. In the beluga whales, plasma lactate started to accumulate after 9-10 min (Shaffer et al., 1997). However, freely diving Weddel seals that have somewhat smaller body size (390 kg) were measured a much longer ADL, 23 min when diving to around 560 m (Williams et al., 2004). The majority of the dives observed here were limited to <10 min (and all <14 min), which could indicate a relatively low ADL compared to other marine mammals (as suggested by Aoki et al., 2017 based on the same data).

**FIGURE 4:**
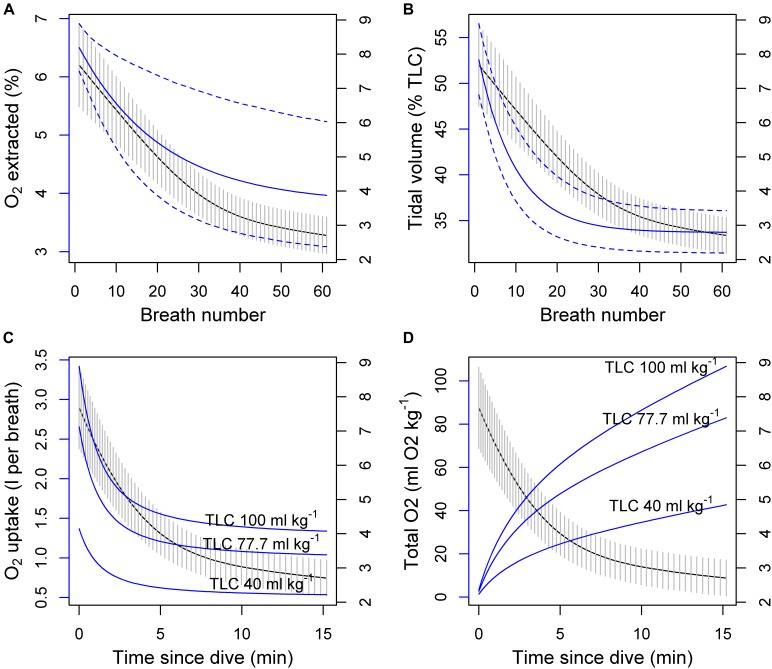
**(A,B)** Potential effects of oxygen extraction rate ΔO_2_ (%) and tidal volume VT (% of total lung capacity, TLC) on the per-breath oxygen consumption (blue line and the left axis) during post-exercise breathing of a hypothetical 1000 kg pilot whale with three alternative TLC values. The black lines (and the right axis) show expected breathing rate from the cumulative model with 95% CI indicated as vertical gray lines. ΔO_2_ and VT measurements and observation error (95% CI in dashed blue lines) were based on measurements in bottlenose dolphins (Fahlman et al., 2016). **(C)** Per-breath uptake (three solid blue lines) was calculated by multiplying the product of ΔO_2_ and VT (top panel solid blue lines) with the three different TLC values. **(D)** Total uptake was then obtained as the sum of the per-breath values over time.

To illustrate how the observed breathing patterns might convert to oxygen consumption, we calculated per-breath oxygen uptake based on the expected breathing pattern following a 10-min dive from the cumulative model, and breath-by-breath estimates of post-exercise oxygen extraction (ΔO_2_, %) and tidal volume (VT, % of total lung capacity, TLC) in bottlenose dolphins (Fahlman et al., 2016) (Figure 4). TLC was calculated for a hypothetical pilot whale with body mass (M_b_) of 1000 kg using the allometric equation 0.135 × M_b0.92_ (Kooyman, 1973; Fahlman et al., 2011), resulting in 77.7 l of air. 95% confidence intervals were constructed using a parametric bootstrap that assumed that each parameter followed a normal distribution with the reported standard deviations (Fahlman et al., 2016). To account for uncertainty in the TLC assumption, the calculation was also made assuming a low TLC value of 40 l (above average estimated diving gas volume 34.6 ml kg^−1^, Aoki et al., 2017) and an upper value of 100 l (rounding up from 95.6 ml kg^−1^, Velten et al., 2013) (Figures 4C,D). Over a hypothetical dive cycle (10 min dive + 10 post-dive), these three values would translate to O_2_ consumption rates of 1.7, 3.4, and 4.3 ml O_2_ kg^−1^ min^−1^, respectively. Using a fixed O_2_ uptake at the end of the 10-min interval (0.6, 1.1, and 1.4 ml O_2_ kg^−1^ per breath) (Figure 4C), and the equivalent post-dive breathing rate from Model 1 (3.4 breaths min^−1^) would have resulted in rates that were 45% lower than estimates based on per-breath consumption. O_2_ consumption rates are likely to be lower outside foraging periods, where breathing rates were estimated to be 2.7–3 breaths min^−1^ (non-recovery rate, Model 3). An average of 3 ml O_2_ kg^−1^ min^−1^ would translate to a daily energy expenditure of 20.7 kCal kg^−1^ day^−1^ (or 86.8 kJ day^−1^; assuming 2.1 J per ml O_2_ and 4.2 J per Cal), which would be consistent with previous energy budget estimates for long-finned pilot whales which were based on 1.2–2x BMR derived from the Kleiber allometric equation (15–25 kCal kg^−1^ day^−1^) (Lockyer, 2007).

Combining the average per-breath O_2_ uptake over the 10-min period (0.75, 1.5, and 1.9 ml O_2_ kg^−1^, Figure 4C) with the apparent net diving cost from Model 2b (1.7 breaths min^−1^) would indicate a diving metabolic rate in long-finned pilot whales to be around 1.3–3.2 ml O_2_ kg^−1^ min^−1^, inclusive of stroking effort. Similarly, the estimated stroking cost of 0.086 breaths could translate to around 0.06–0.16 ml O_2_ kg^−1^ (1.3–3.2 J kg^−1^) which is similar to previously estimated cost of stroking in beluga whales when swimming at preferred swim speeds (2.2 J kg^−1^, Williams et al., 2017) and in freely diving Weddel seals (2.4 J kg^−1^, Williams et al., 2004). An average fluke rate of 18.3 strokes min^−1^ during >31 m dives would therefore cost 1.2–2.9 ml O_2_ kg^−1^ min^−1^, or around 92% of the net diving cost. While the net diving cost appears small relative to the total body O_2_ stores estimated for pilot whales, only a portion of the total O_2_ is stored in the muscle (42% of the total, or 29 ml O_2_ kg^−1^, Velten et al., 2013). Thus the relatively low net diving cost, largely determined by locomotion effort, may indicate that the 10-min foraging dives are limited by the muscle store rather than the total O_2_ store, or the capacity of the muscle to store and tolerate CO_2_. Such scenario would be plausible if blood flow was restricted to the muscle to maintain O_2_ supply elsewhere, such as the central nervous system (Ponganis, 2015).

These speculative interpretations highlight the importance of physiological measurements of ΔO_2_, VT, and end-tidal CO_2_. In particular, the calculations assume that the variation of ΔO_2_ and VT is similar following a 10-min swimming exercise in bottlenose dolphins, vs. 10-min foraging dives in long-finned pilot whales. Measurements of ΔO_2_ and VT would be especially useful to have for a range of activity contexts (such as following dives of different duration) so that they would be more directly applicable to calculating energy budgets of free-ranging animals.

### Methods Perspectives

We developed a new cumulative dive history model to estimate apparent diving costs and recovery time from all previous dives, including both shallow and deep diving periods. The model may be applied to any breath-hold divers, and could also be extended to terrestrial mammals with distinct bouts of exercise, such as prey capture attempts. In humans, post-exercise recovery has been linked to exercise intensity and duration (Børsheim and Bahr, 2003), and so changes in the recovery pattern, such as dyspnea or increased recovery time, could be used to indicate impaired circulatory response (e.g., Cohen-Solal et al., 1995). One key assumption of the model is that any serial correlation of the time series is captured by the time-decay in breathing rate over time. Therefore, as a cautionary approach, we chose to test the effects of sound exposures in a GAMM using the expected breathing rate from the cumulative model as an explanatory variable. Even this approach did not completely remedy serial correlation in the model residuals. However, the cumulative model does have potential to incorporate disturbance effects on breathing rate, and separate those effects in terms of net diving costs vs. immediate changes in breathing rate (non-recovery rate). Indeed, the specific cumulative model structure which formed the basis of the new dive history model was developed to detect behavioral responses of Cuvier’s beaked whales to navy sonar (DeRuiter et al., 2013).

The cumulative model fitted the data well despite the lack of an explicit physiological mechanism for the effects of repeated dives on gas exchange. Specifically, the model assumes that breathing rate recovers as a function of time since dive, and not the number of breaths. In other words, the effect of previous dives on breathing rate continued to decay even when the animal was not exchanging gas at surface. This is a reasonable assumption for aerobic exercise, but for a breath-hold diver a more physiologically complete model would incorporate per-breath O_2_ loading and O_2_ debt (e.g., Roos et al., 2016); however, the explanatory power of a sole O_2_ model could be limited if CO_2_ instead is driving the surface behavior.

We did not include stroking amplitude in our metric for locomotion effort, only stroke numbers, which may have caused additional variation in the estimated relationship with breathing rate. It would be interesting to compare whether other metrics for locomotion effort (e.g., amplitude-corrected stroking effort, dynamic body acceleration) would improve the relationship between locomotion costs and breathing effort. However, we caution against simple correlations between total energy expenditure and sum of accelerometer-based values (such as total fluke strokes, or total dynamic acceleration). Because time is included in both sides of such an equation, a bivariate correlation alone is not sufficient to show that acceleration is predictive of metabolic rate (so called ‘time trap’; Halsey, 2017; Ladds et al., 2017). Our analysis allowed both dive duration and fluke strokes to be included as additive covariates in the same model, and thus explicitly treated the effect of time (‘basal diving cost’) and locomotion effort separately. Furthermore, Model 2 model selection indicated total stroking effort, rather than basal rates (dive duration) and/or intensity of fluking (fluke stroke rate) predicted post-dive number of breaths. However, a larger dataset with a greater variety of deep diving behaviors could help to more clearly separate the effects of total diving vs. total locomotion effort, as the two are inherently correlated.

## Data Availability Statement

The datasets that were analyzed and generated in this manuscript are enclosed with the manuscript as Supplementary Material, along with a data report and all R-code for conducting the presented analyses.

## Author Contributions

SI and PM conceived the conceptual approach of the manuscript. PM, PK, and CC designed the field data collection as part of the 3S project. KA and SI processed the tag data. SI designed and performed the statistical analyses, and drafted the manuscript. All authors contributed to the field data collection, discussed the results, and commented on the manuscript.

## Conflict of Interest Statement

The authors declare that the research was conducted in the absence of any commercial or financial relationships that could be construed as a potential conflict of interest.
