# Supplementary material for: Breathing Patterns Indicate Cost of Exercise During Diving and Response to Experimental Sound Exposures in Long-Finned Pilot Whales
Source: Front Physiol. 2018 Oct 25;9:1462. doi: 10.3389/fphys.2018.01462 (PMC6232938; doi:10.3389/fphys.2018.01462)
Supplement: Supplementary file 1 [file Table_1.DOCX]

#### Appendix A. Supplementary Figures


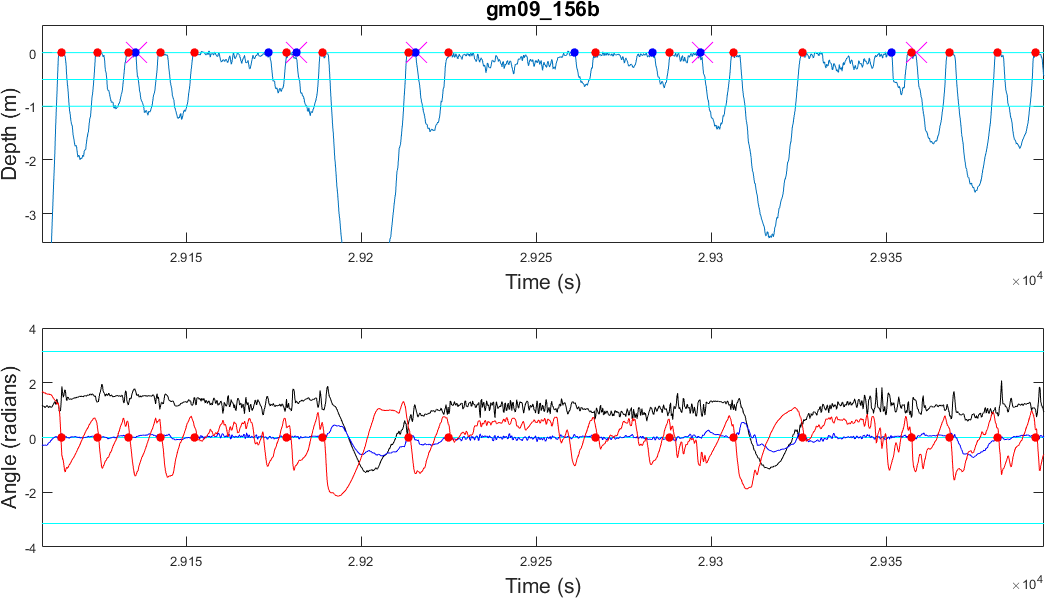


Figure A1. Example time series plots for marking breath times. Top panel: dive profile (dark blue) overlaid with automatically and manually marked breath times (red circles). The single-breath surfacings were characterized by an arch in the pitch signature near the sea surface. Breath time was deﬁned at the point at which the pitch was brieﬂy horizontal. Visits near the sea surface with roll other than zero were not marked as breaths, and instead were considered part of the breath-holding interval between breaths. Near-surface behaviors with uncertainty about the number or timing of breaths were marked as "surface intervals". The end of these was marked manually (blue circles, not considered as breaths). Pink crosses indicate when the automatically detected surface intervals exceed >2s in duration, as a guide for the manual marking. Horizontal cyan lines are reference depths at 0, 0.5 and 1 meters. The inter-breath-interval (IBI, s) and dive duration (s) was measured as the time period between the last breath (red) or the end of surface period (blue) and the next breath or start of surface period. Each breath was associated with the 5 Hz data time series by assigning it to the closest two data samples, which resulted in minimum breath duration of 0.2s. Bottom panel: pitch (red), roll (blue) and heading (black) in radians. Breath time was deﬁned at the point at which the pitch was brieﬂy horizontal; also note variation in roll when the animal is not taking a breath at surface. Horizontal cyan lines mark ± π.

Figure A2. Histogram of inter-breath-intervals (IBI) based on breaths heard on the tag acoustic record.


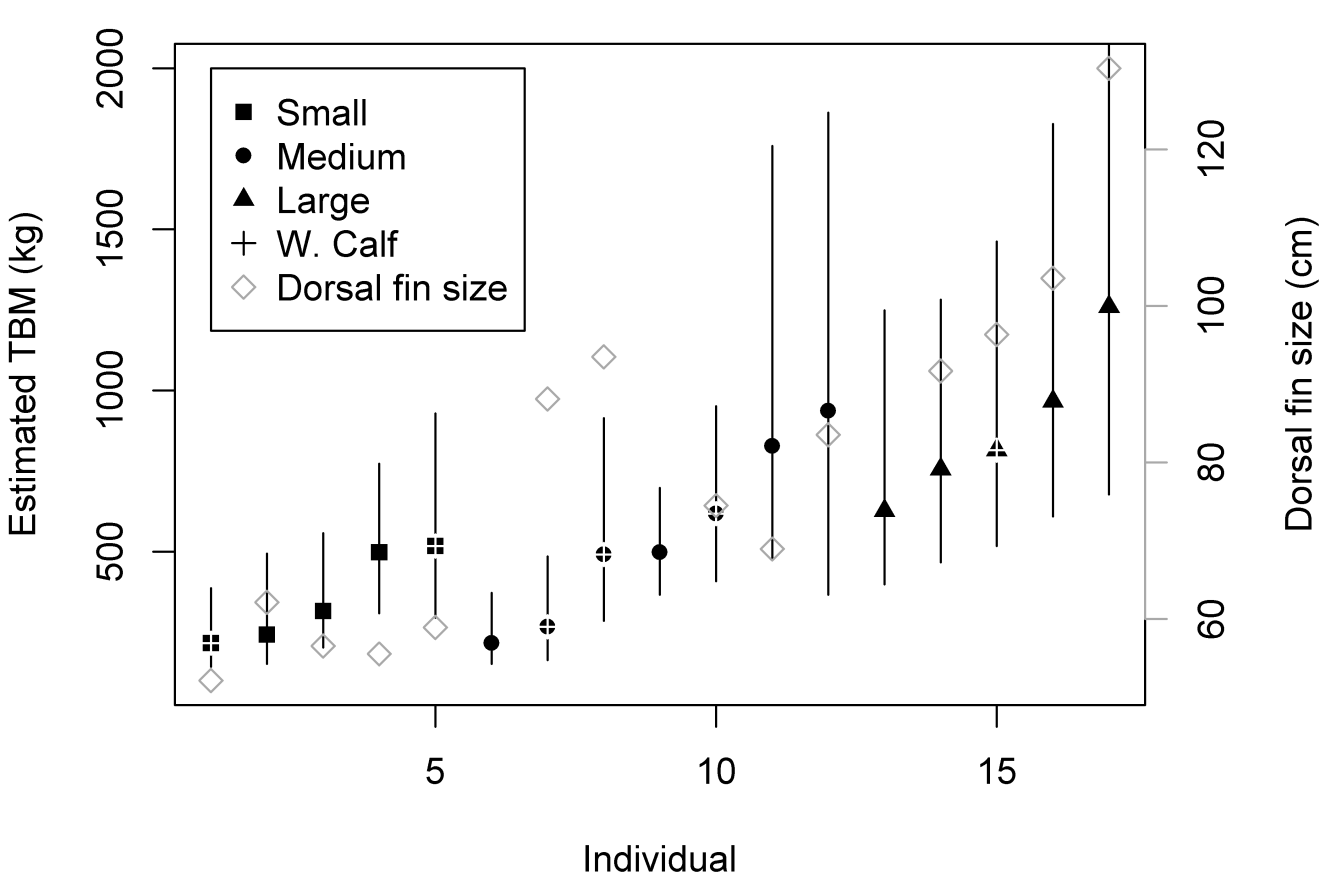


Figure A3. Individual size classification and association with a calf. Body size class was determined by combining field estimates (small/medium/large adult), and where available, estimates of dorsal fin size from good quality photographs of the tag attached to the dorsal fin of the whale. The base of the dorsal fin (Augusto et al 2013) was measured in perpendicular photographs, and scaled to known length of the tag. Two field-estimated ‘medium’ animals were re-classed to ‘small’ (gm08_150c, gm10_152b), and one ‘large’ animal was re-classed as ‘medium’ (gm14_180b). In the resulting classification, the base of the dorsal fin was estimated 52-62 cm in small animals, 69-93 cm in medium animals, and 92-130 cm in large animals. Body mass was derived from fundamental stroking frequency using the equation $exp(\frac{log(\frac{fs}{3.56})}{-0.29})$ following Sato et al 2006. Vertical lines show body mass estimates based upon the half width of the fundamental frequency peak. Not all tagged whales had good quality photographs, and so dorsal fin size was not estimated.


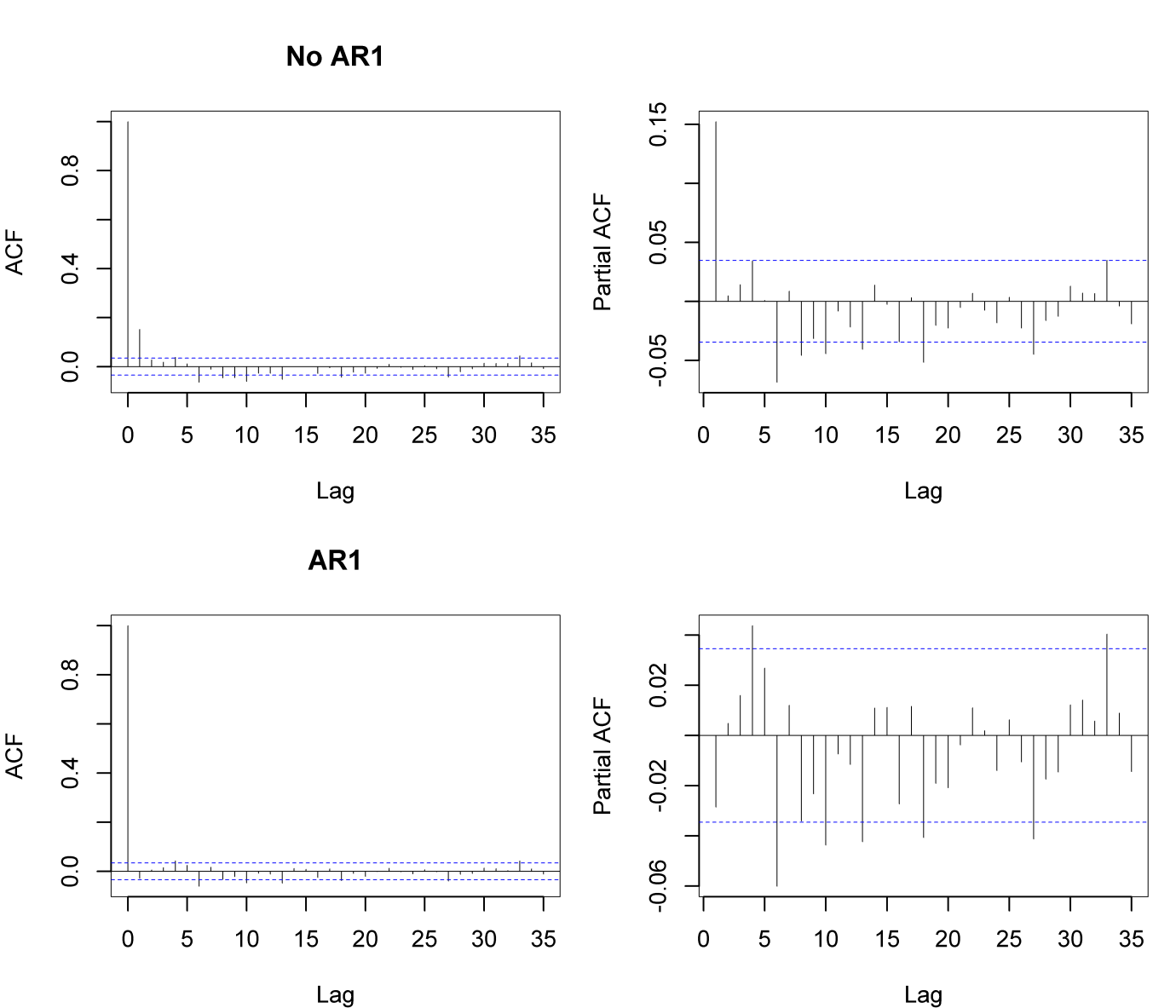
Figure A4. Autocorrelation function (ACF) (left panels) and partial ACF (right panels) of Model 1 residuals when excluding (top panels) and including (bottom panels) 1st order autoregressive correlation (AR1) structure.


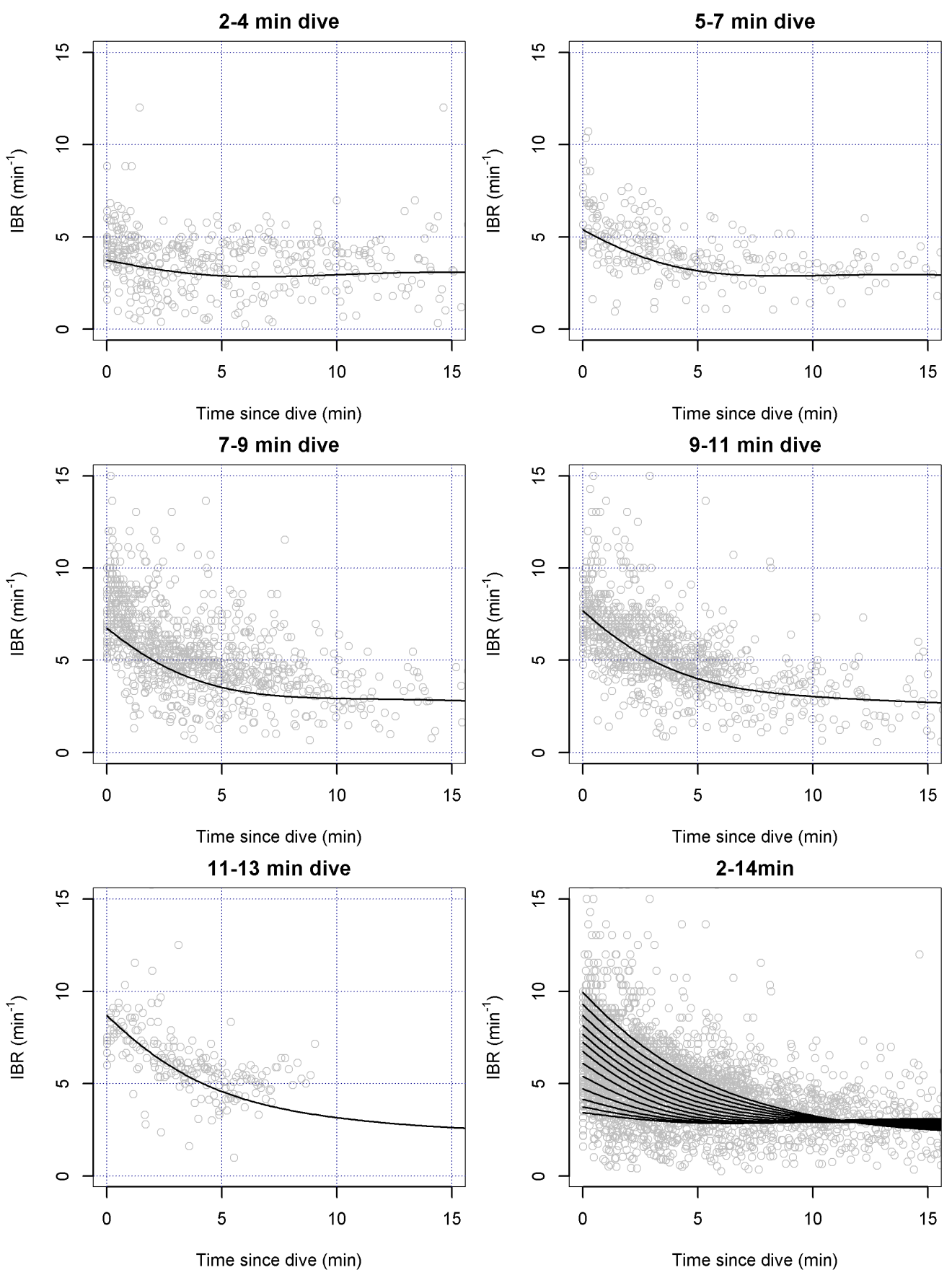


Figure A5. Model predictions (instantaneous breathing rate [IBR, min^-1^] from Model 1


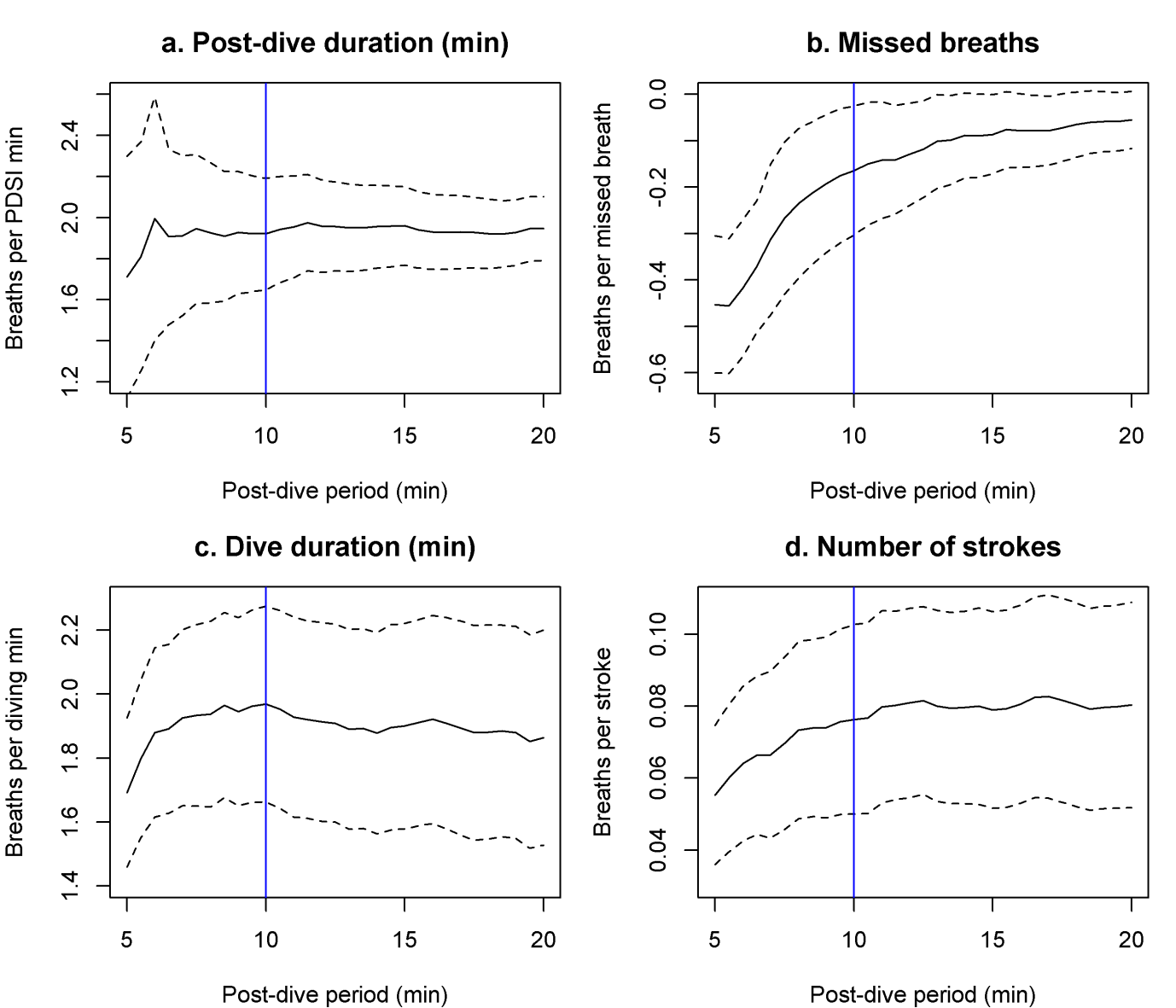


Figure A6. Sensitivity of parameter estimates (y-axis) on the selection of post-dive window duration over which to calculate number of breaths (x-axis) for Model 2. Stroking effort was not included in the model to generate estimates for panels a-c. Thus, the estimated breaths per dive duration (i.e. dive cost, min^-1^) includes any locomotory effort. Panel d model included number of strokes and the three main effects (PDSI duration, missed breaths, and dive duration).


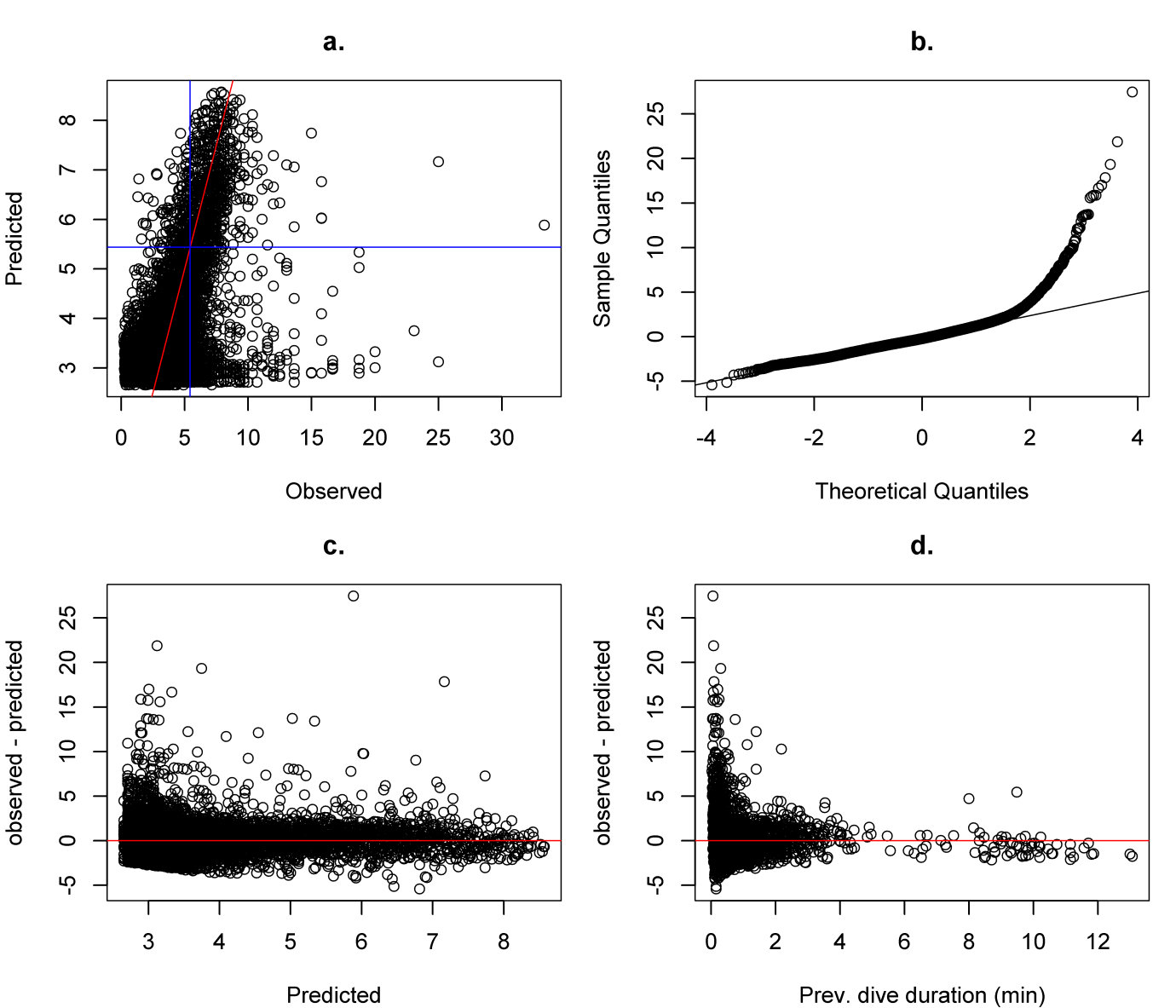


Figure A7. Residual plots of the best cumulative model (Model 3). Panel a: predicted values as a function of observed values; red line shows one-to-one line, and blue lines show estimated maximum additive breathing rate. Panel b: Normal q-q-plot. Panel c: raw residuals (observed – predicted) are against predicted values. Panel d: raw residuals plotted against previous dive duration.


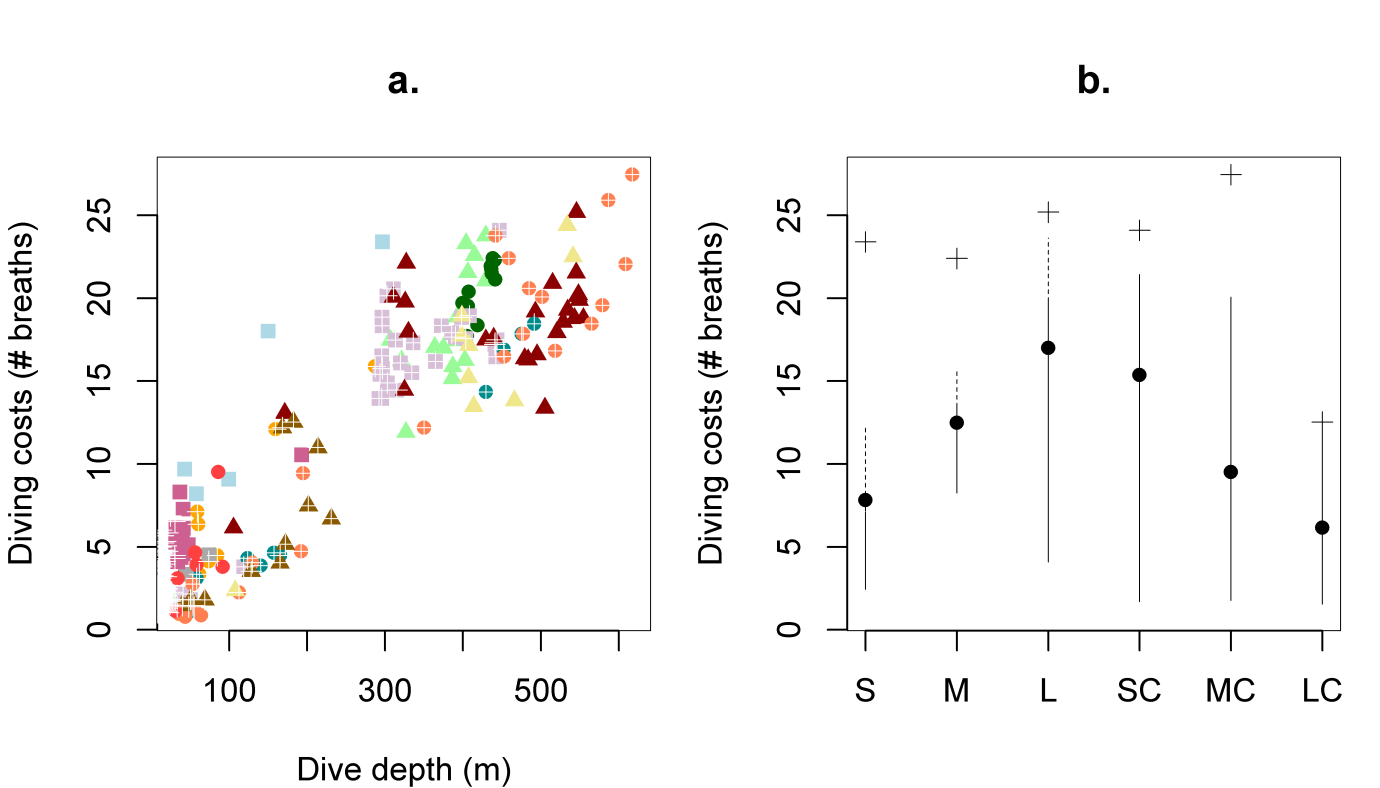


Figure A8. Model 3 estimated diving costs for each observed dive (>31m), as a function of maximum dive depth (panel a), and stratified by individual type (panel b). Panel a: Symbols indicate individual type (square: small body size, triangle: large body size, crosses: calf association), while colours indicate different individuals. Panel b: Crosses show maximum values for each individual category (S-small, M-medium, L-large, C-calf). Individual mean values (solid circles) and 95% quantiles (lines) were averaged for each individual category. Solid lines show diving costs excluding stroking effort.


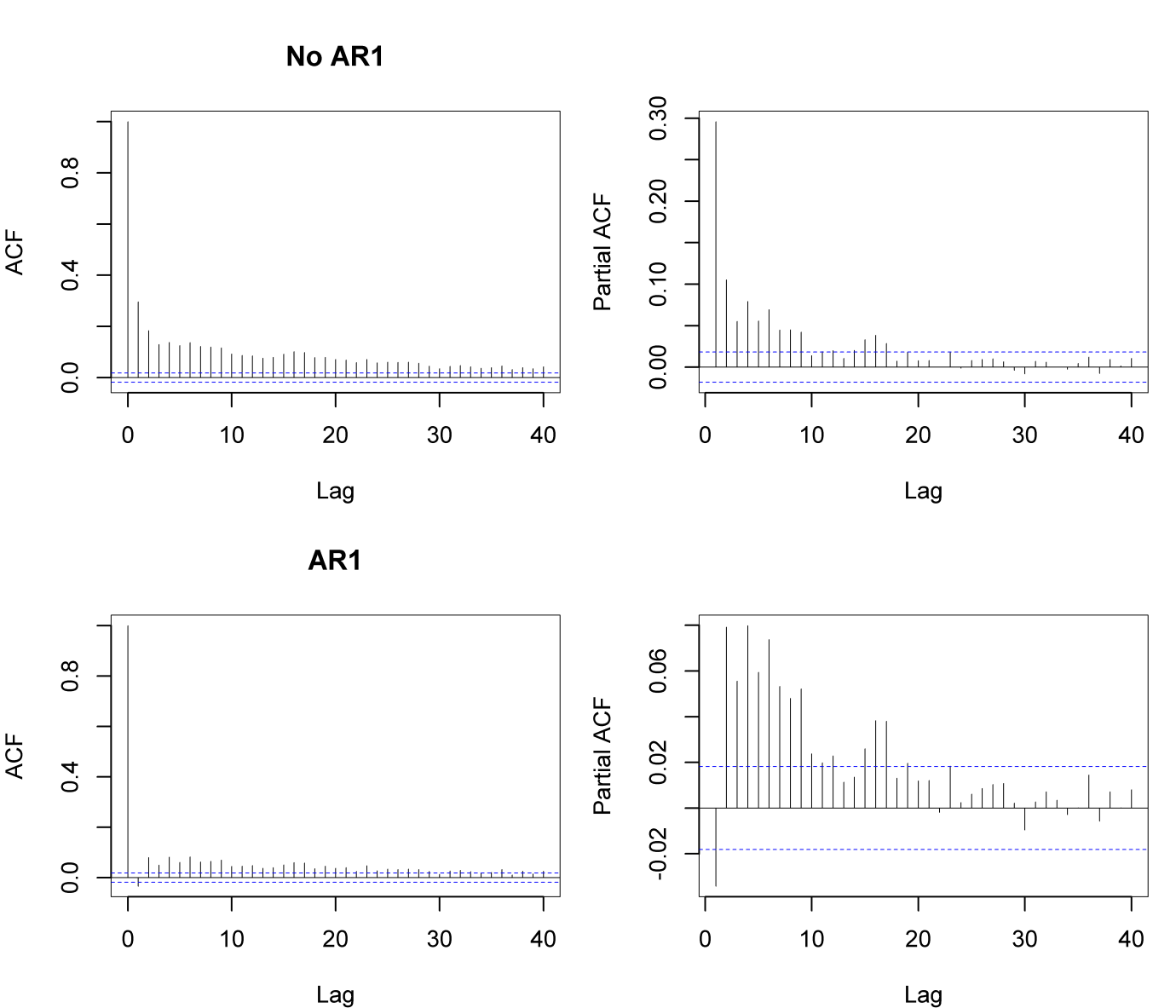


Figure A9. Autocorrelation function (ACF) (left panels) and partial ACF (right panels) of the Model 4 residuals when excluding (top panels) and including (bottom panels) 1st order autoregressive correlation (AR1) structure.


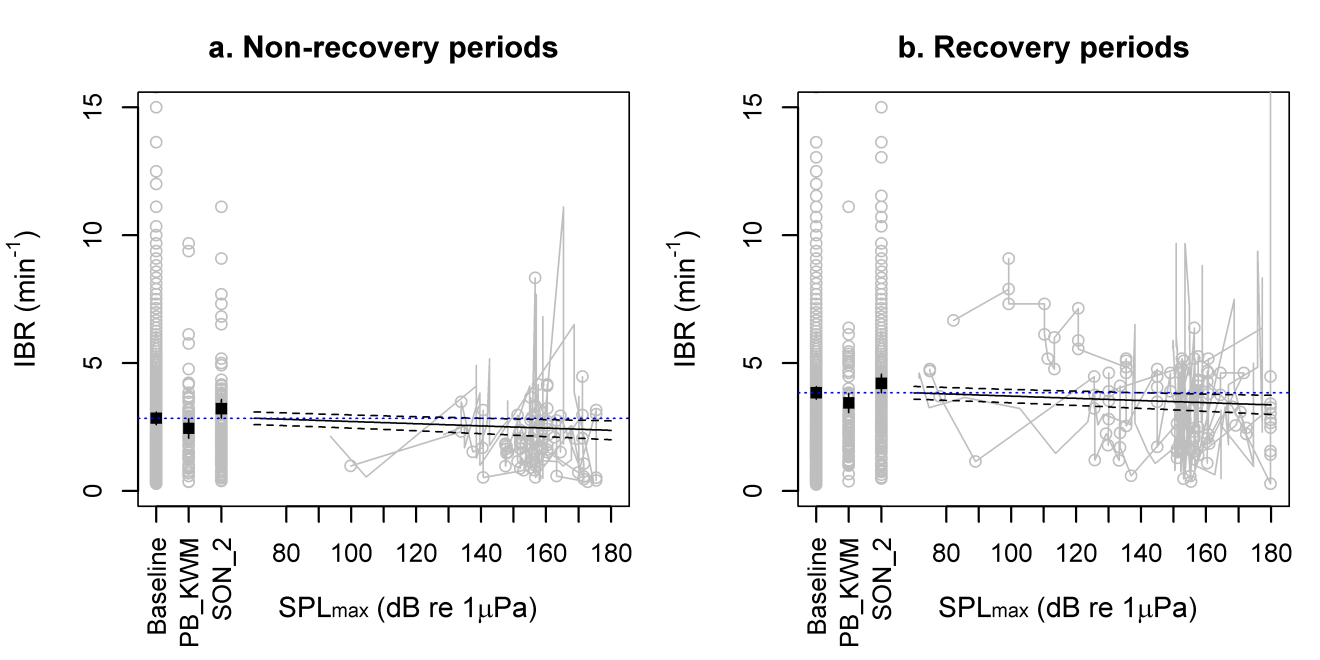


Figure A10. Observed (grey) and Model 4 predicted (black) instantaneous breathing rates (IBR; above rate expected by Model 3) are shown during pre-exposure baseline, as a function of received level (SPL_max_) during the first sonar approach, during subsequent sonar approaches (SON_2) and during mammal-feeding killer whale playbacks (PB_KWM). Non-recovery (Panel a) vs. recovery periods (Panel b) were defined as below or above 110% of the non-recovery baseline rate estimated by the cumulative model for each individual type. Horizontal dotted lines show baseline values in each case.

Table A1. Increase in source level (‘ramp-up’) during the first 10 min of MFAS and LFAS exposure sessions

| Time (hh:mm:ss) | Source level (dB re 1 μPa m) | |
| --- | --- | --- |
|  | 1-2 kHz (LFAS) | 6-7 kHz (MFAS) |
| 00:00:00 | 152 | 158 |
| 00:00:20 | 155 | 160 |
| 00:00:40 | 166 | 162 |
| 00:01:00 | 183 | 164 |
| 00:01:20 | 194 | 166 |
| 00:01:40 | 195 | 168 |
| 00:02:00 | 197 | 170 |
| 00:02:20 | 198 | 172 |
| 00:02:40 | 199 | 174 |
| 00:03:00 | 200 | 176 |
| 00:03:20 | 201 | 178 |
| 00:03:40 | 202 | 180 |
| 00:04:00 | 202 | 182 |
| 00:04:20 | 203 | 184 |
| 00:04:40 | 204 | 186 |
| 00:05:00 | 204 | 188 |
| 00:05:20 | 205 | 190 |
| 00:05:40 | 205 | 191 |
| 00:06:00 | 206 | 192 |
| 00:06:20 | 206 | 193 |
| 00:06:40 | 207 | 194 |
| 00:07:00 | 208 | 195 |
| 00:07:20 | 209 | 196 |
| 00:07:40 | 210 | 196 |
| 00:08:00 | 211 | 197 |
| 00:08:20 | 212 | 197 |
| 00:08:40 | 212 | 198 |
| 00:09:00 | 213 | 198 |
| 00:09:20 | 213 | 198 |
| 00:09:40 | 213 | 199 |
| 00:10:00 | 214 | 199 |
