# Supplementary material for: Breathing Patterns Indicate Cost of Exercise During Diving and Response to Experimental Sound Exposures in Long-Finned Pilot Whales
Source: Front Physiol. 2018 Oct 25;9:1462. doi: 10.3389/fphys.2018.01462 (PMC6232938; doi:10.3389/fphys.2018.01462)
Supplement: Supplementary file 2 [file Table_2.DOCX]

#### Appendix B. Cumulative model details

The cumulative model quantified dive recovery as an elevated post-dive breathing rate that exponentially approaches a level $\beta_{i}$ that is no longer influenced by previous diving history. The level was allowed to vary between individual size classes and between individuals with and without a calf in a linear regression:

$$\beta_{i}=exp\left( \beta_{0}+ \beta_{1}*{calf}_{i}+ \beta_{2}*{small}_{i}+ \beta_{3}*{large}_{i} \right) Eq. 1$$

Where $\beta_{i}$ is the baseline breathing rate for individual $i$, $\beta_{0}$ is the intercept and $\beta_{1}$, $\beta_{2}$, $\beta_{3}$ coefficients associated with the respective presence/absence covariates *calf*, *small*, and *large*.

Net diving costs $\theta_{k}$ were expressed as the total number of breaths required to recover from a dive:

$$\theta_{k}=\delta_{i}*{dive.dur}_{k}+ \varphi*{fluke.no}_{k} Eq. 2$$

Where $\delta_{i}$ is the basal diving cost (number of breaths incurred due to diving, min^-1^) for each individual $i$ and $\varphi$ is the individual-average locomotion cost (number of breaths incurred per fluke stroke) for each dive $k$. As with baseline rate, basal diving cost $\delta_{i}$ was modeled as a linear regression with body size class and individual association with a calf as candidate covariates.

Breathing rate was modeled as the sum of the baseline rate $\beta_{i}$ and the exponentially decaying net diving costs of all previous dives:

$$E\left( {ibr}_{k} \right)= \beta_{i}+ \sum_{k=1}^{K_{i}} f_{k}\left( t_{k} \right)$$

$$f_{k}\left( t_{k} \right)=\left\{ \begin{aligned} {\rho_{k}*e}^{(\frac{-t_{k}}{\tau})}, if t_{k}> 0, \\ 0, otherwise \end{aligned} \right. Eq. 3$$

Where $E\left( ibr \right)$ is the expected value of instantaneous breathing rate, $\beta_{i}$ is the baseline breathing rate for individual $i$, $K_{i}$ is the number of dives for an individual $i$ and $t_{k}$ is the elapsed time since dive $k$. The exponential decay was expressed in terms of mean lifetime $\tau_{k}$, i.e. the time at which breathing rate is expected to reduce to 1/e (~37%) of the initial breathing rate $\rho_{k}$.

The decay function was estimated for net diving costs that exceeded a single breath. Thus, the initial elevated breathing rate and the subsequent decay following a single dive $k$ was modeled as a function of time since the first breath was taken at the end of that dive. Let $n_{k}$ denote the number of subsequent breaths required to recover from dive $k$, calculated in the model using a step function, ${max(\theta}_{k}-1,0)$. The initial breathing rate $\rho_{k}$was assumed to increase linearly with $n$ up to a maximum $\rho_{max}$ rate (min^-1^):

$$\rho_{k}=min(r*n_{k}, \rho_{max}) Eq. 4$$

Where $r$ is a positive valued parameter describing the increase in initial breathing rate (min^-1^) for every additional breath required to recover from dive $k$.

To express the total number of remaining breaths under the decay function, the decay function was integrated as follows:

$$n_{i,k}=\int_{0}^{\infty} {\rho_{i,k}*e}^{(\frac{-t_{k}}{\tau})}=\lim_{t\to\infty} \left( -\rho_{i,k}*\tau*e^{\left( \frac{-t_{k}}{\tau} \right)} \right)+C-\left( -{\rho_{i,k}*\tau*e}^{\left( \frac{-0}{\tau} \right)} \right)-C$$

$$=-\rho_{i,k}*\tau*0+\rho_{i,k}*\tau*1=\rho_{i,k}*\tau Eq. 5$$

This expression allowed us to calculate mean lifetime $\tau$ as $\frac{n_{i,k}}{\rho_{i,k}}$ and parameterize the decay function in terms of $n$:

$$f_{k}\left( t_{k} \right)=\left\{ \begin{aligned} {\min\left( r*n_{i,k}, \rho_{max} \right)*e}^{\left( \frac{-t_{k}*\rho_{i,k}}{n_{i,k}} \right)}, if t_{k}> 0, \\ 0, otherwise \end{aligned} \right.$$

$$f_{k}\left( t_{k} \right)=\left\{ \begin{aligned} {r*n_{i,k}*e}^{\left( -t_{k}*r \right)} if {p_{i,k}\leq\rho_{max} and t}_{k}> 0 \\ {\rho_{max}*e}^{\left( \frac{-t_{k}*\rho_{max}}{n_{i,k}} \right)} if {p_{i,k}>\rho_{max} and t}_{k}> 0, \\ 0, otherwise \end{aligned} \right. Eq. 6$$

Breathing rate was specified a gamma distribution. The distribution was estimated a variance parameter $\epsilon$ and the likelihood calculated following DeRuiter et al (2013). The negative log-likelihood was minimized using the mle2 in r (package bbmle).

The model was fitted to non-exposure data only, and then used to predict breathing rates for all of the data, including exposures. Similar to the other models, only shallow inter-breath intervals (<31m) were considered to be of interest for modeling breathing behavior and so all ≥31m dives were excluded from the response data. However, unlike Models 1-2, all previous inter-breath intervals, regardless of their depth, were allowed to contribute to the net diving cost $\theta_{k}$ which contributed in the model as an explanatory variable.

The models were fitted six times each with different initial values and the stability of the resulting likelihoods was monitored visually. Initial values for the intercepts ($\beta_{0}$, $\delta_{0}$, and $\varphi_{0}$) were drawn randomly from a normal distribution (mean=0, sd=1), while the initial values for the individual-specific coefficients ($\beta_{1}$, $\beta_{2}$,$\beta_{3}$,$\delta_{1}$, $\delta_{2}$,$\delta_{3}$) were fixed to 0. Initial values for positive-valued parameters were drawn from gamma distribution ($\epsilon$, $r$) and a uniform distribution ($\rho_{max}$).

Please see attached R script (*Step4_Model_3.R*) for further details about the model specification and fitting. To fit the models, unpack the supplementary zip file into your working directory in R (including all .csv, .R and .Rd files), and run the R script following in-line documentation. R data storage files (.Rd) can only opened in R using the load() command.
