# Supplementary material for: Breathing Patterns Indicate Cost of Exercise During Diving and Response to Experimental Sound Exposures in Long-Finned Pilot Whales
Source: Front Physiol. 2018 Oct 25;9:1462. doi: 10.3389/fphys.2018.01462 (PMC6232938; doi:10.3389/fphys.2018.01462)
Supplement: Supplementary file 3 [file Table_3.docx]

#### Appendix C. All dive profiles and cumulative model predictions


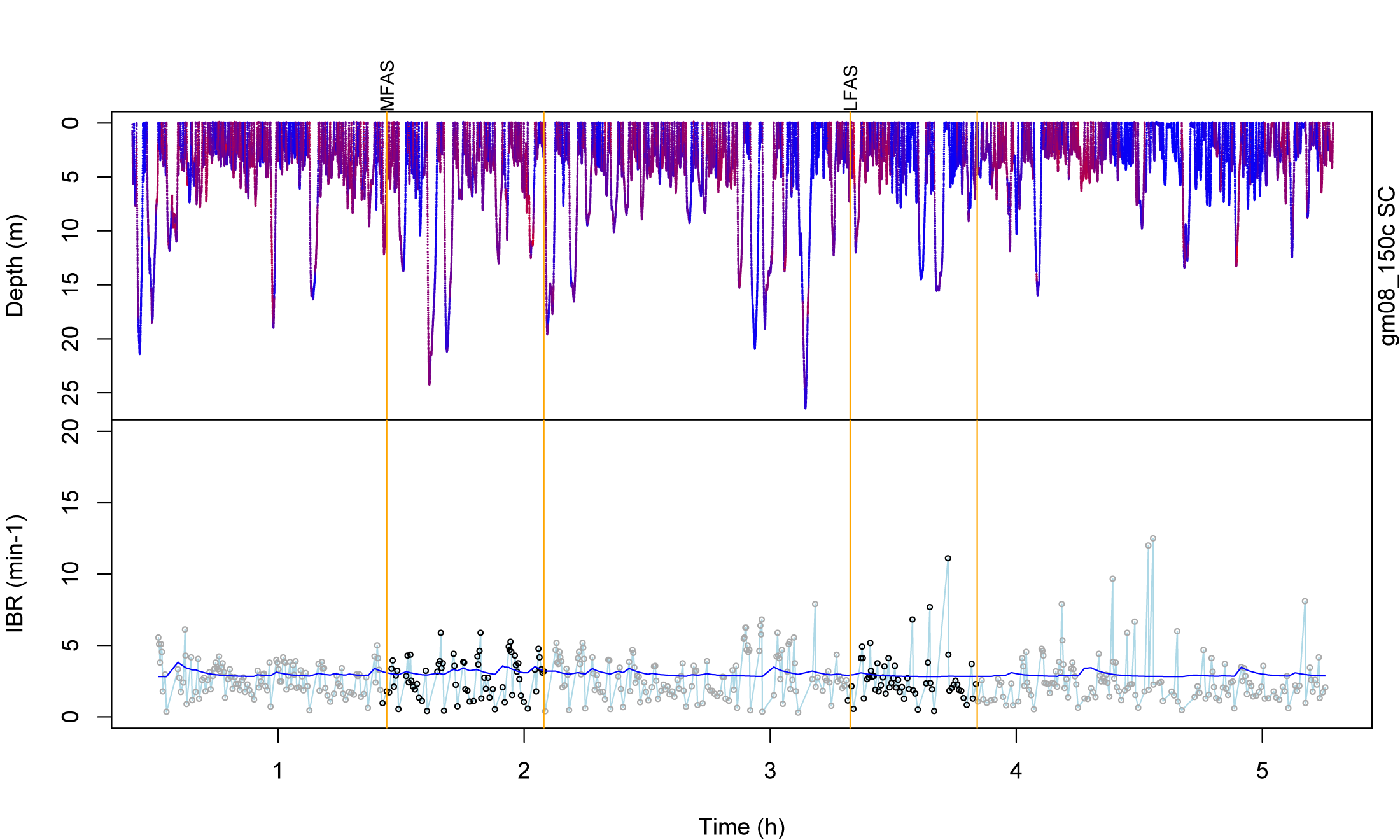


Fig. C1 Top panel shows the dive profile, color-coded by fluke stroke rate (red: higher rate). Orange vertical lines show sound exposure start and end times (MFAS: medium-frequency active sonar, LFAS: low-frequency active sonar). Bottom panel shows instantaneous breathing rate (IBR, connected circles) overlaid with the cumulative model estimates (dark blue line). Black circles show IBR values not included in the cumulative model fitting. Tag deployment code is shown on the right, with individual class (SC: Small body size, associated with a calf).


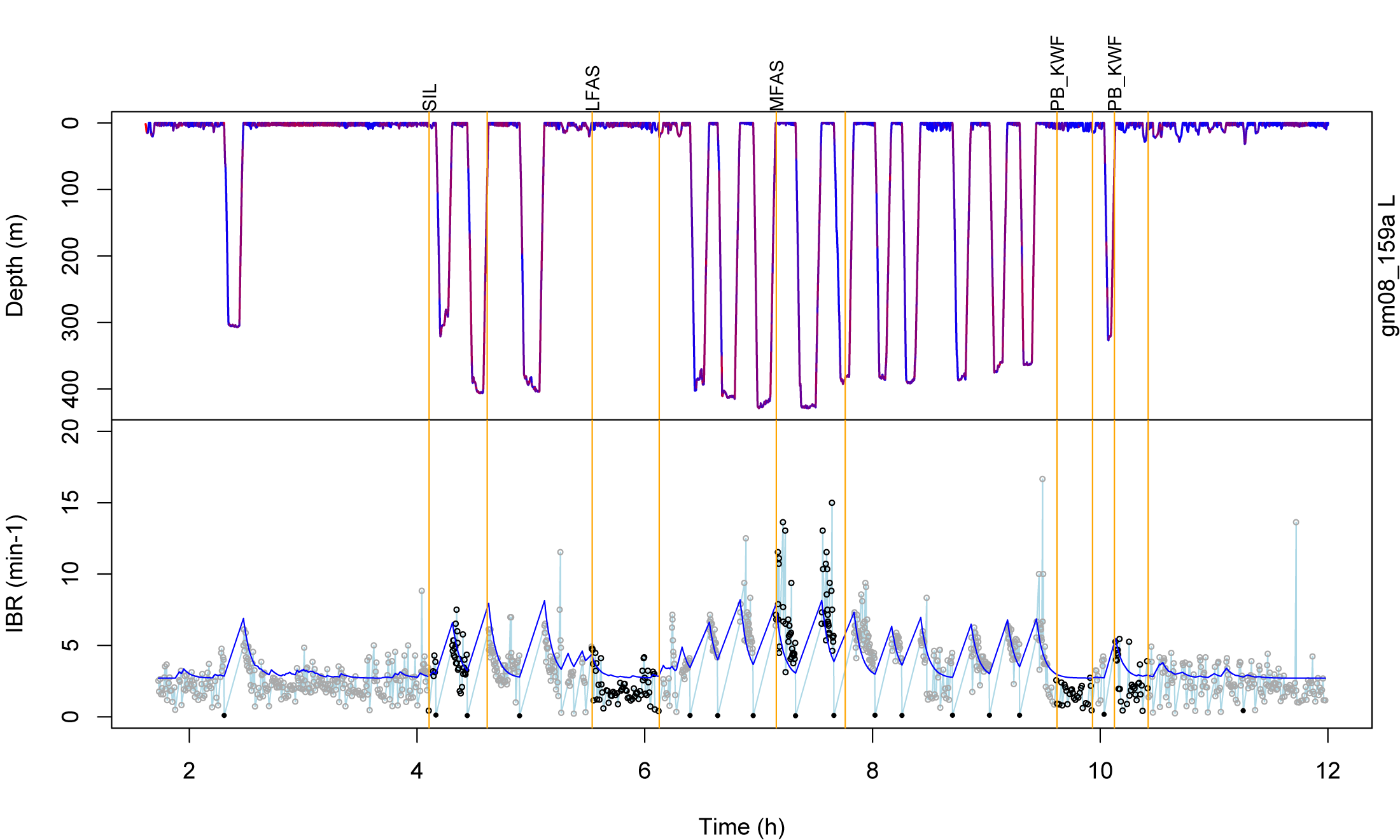


Fig. C2 Top panel shows the dive profile, color-coded by fluke stroke rate (red: higher rate). Orange vertical lines show sound exposure start and end times (SIL: no-sonar approach, LFAS: low-frequency active sonar, MFAS: medium-frequency active sonar, PB_KWF: fish-eating killer whale sound playback). Bottom panel shows instantaneous breathing rate (IBR, connected circles) overlaid with the cumulative model estimates (dark blue line). Black circles show IBR values not included in the cumulative model fitting. Tag deployment code is shown on the right, with body size class (L: Large).


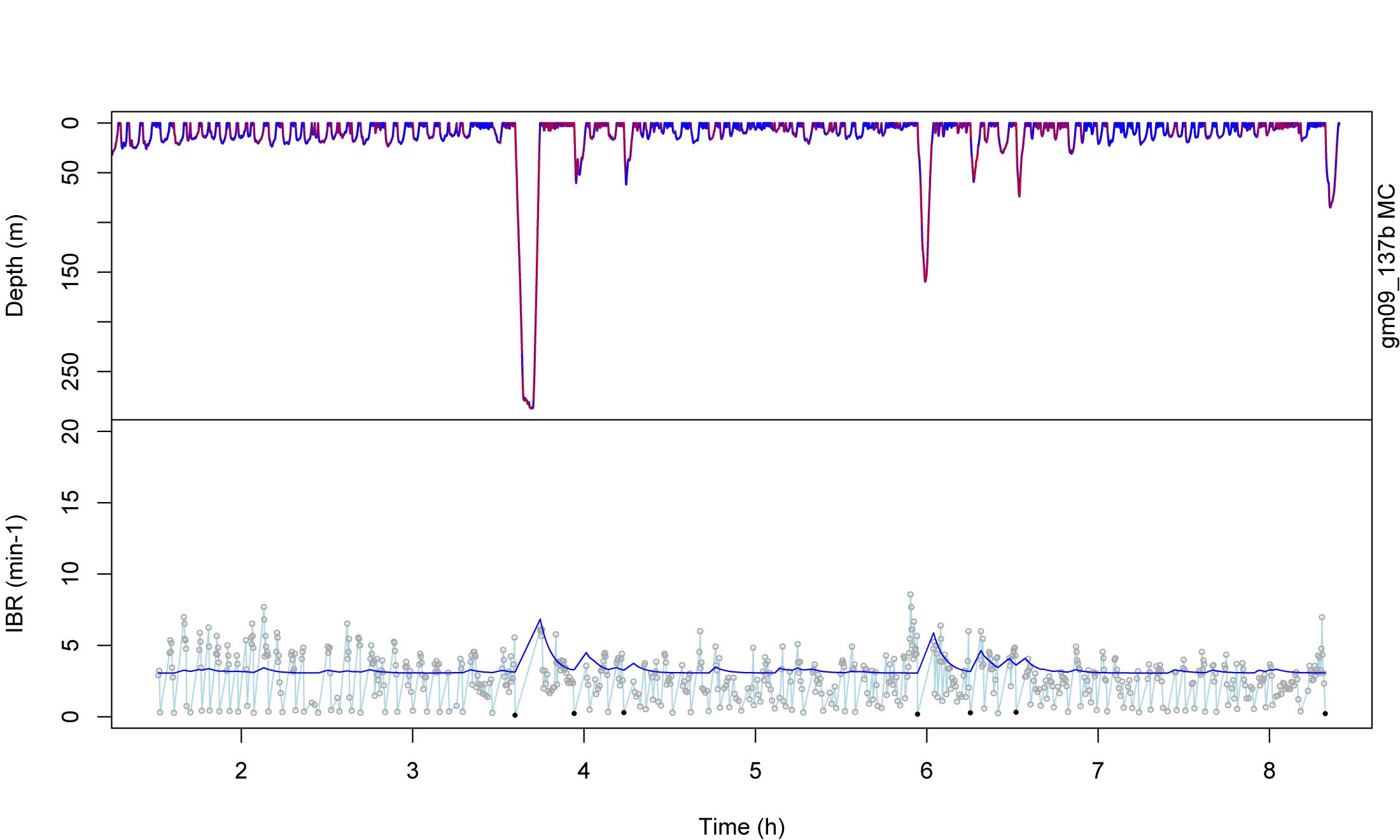


Fig. C3 Top panel shows the dive profile, color-coded by fluke stroke rate (red: higher rate). Bottom panel shows instantaneous breathing rate (IBR, connected circles) overlaid with the cumulative model estimates (dark blue line). Black circles show IBR values not included in the cumulative model fitting. Tag deployment code is shown on the right, with individual class (MC: Medium body size, associated with a calf).


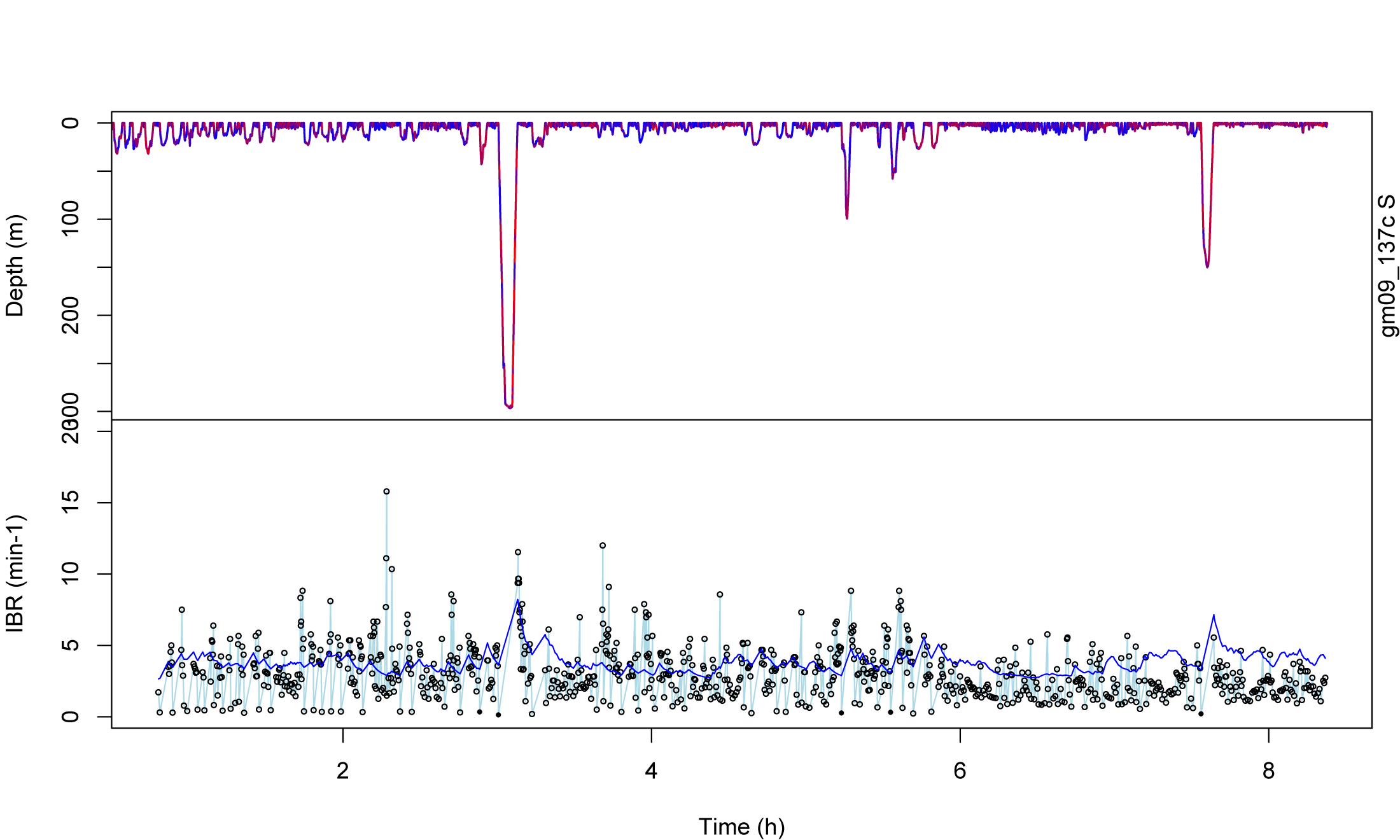


Fig. C4 Top panel shows the dive profile, color-coded by fluke stroke rate (red: higher rate). Bottom panel shows instantaneous breathing rate (IBR, connected circles) overlaid with the cumulative model estimates (dark blue line). Black circles show IBR values not included in the cumulative model fitting. Tag deployment code is shown on the right, with body size class (S: Small).


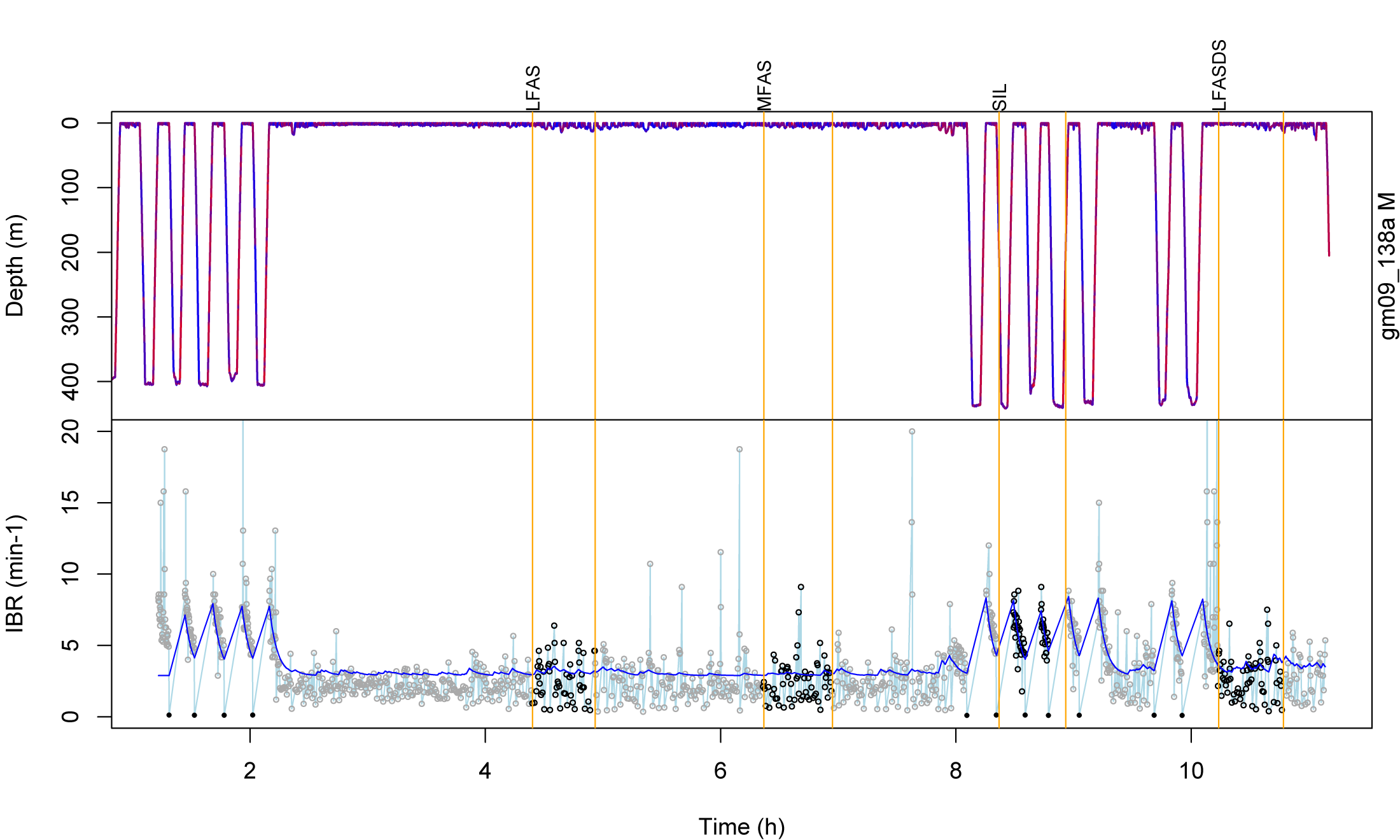


Fig. C5 Top panel shows the dive profile, color-coded by fluke stroke rate (red: higher rate). Orange vertical lines show sound exposure start and end times (LFAS: low-frequency active sonar, MFAS: medium-frequency active sonar, SIL: no-sonar approach, LFAS: low-frequency active sonar down-sweep signal). Bottom panel shows instantaneous breathing rate (IBR, connected circles) overlaid with the cumulative model estimates (dark blue line). Black circles show IBR values not included in the cumulative model fitting. Tag deployment code is shown on the right, with size class (M: Medium).


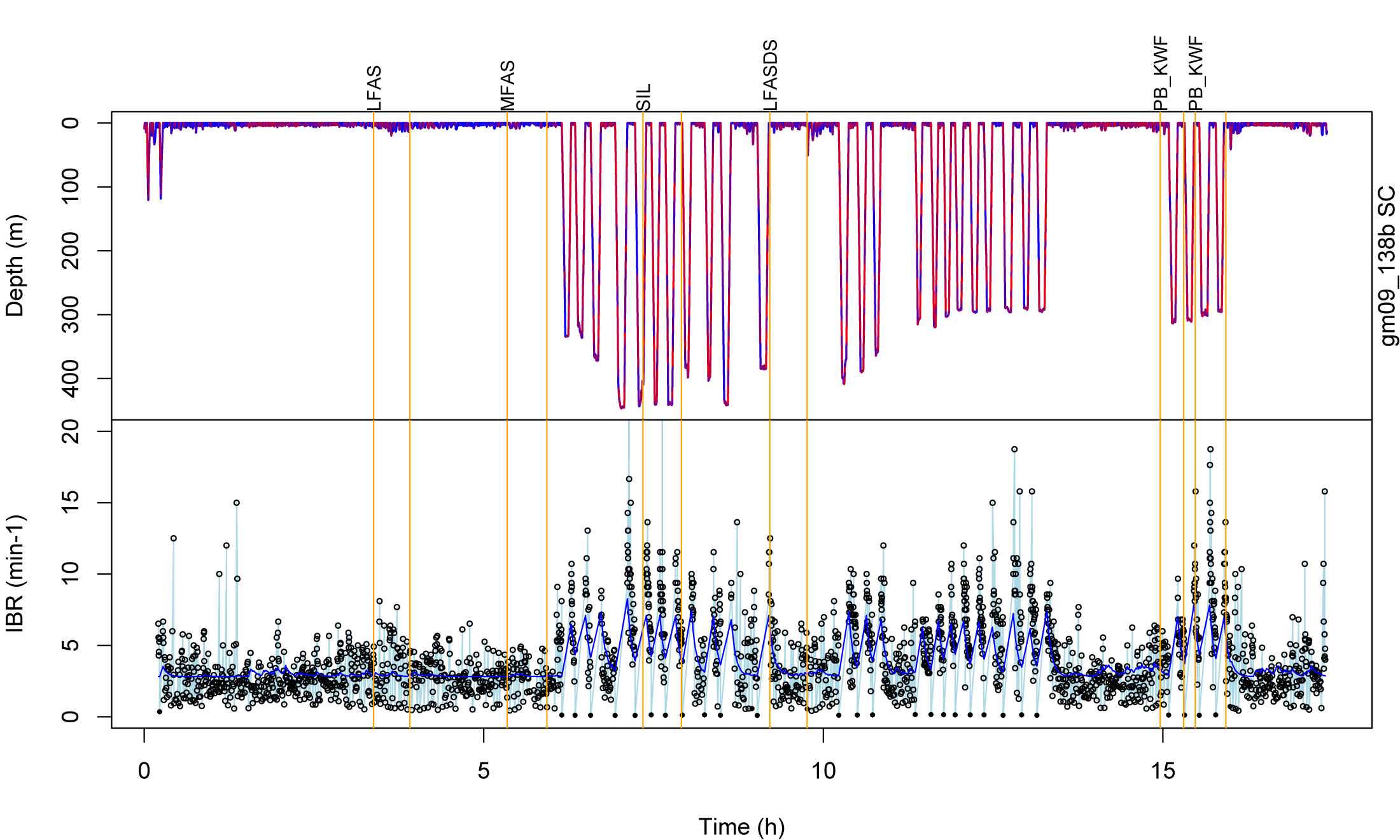


Fig. C6 Top panel shows the dive profile, color-coded by fluke stroke rate (red: higher rate). Orange vertical lines show sound exposure start and end times (LFAS: low-frequency active sonar, MFAS: medium-frequency active sonar, SIL: no-sonar approach, LFASDS: low-frequency active sonar down-sweep signal, PB_KWF: fish-eating killer whale sound playback). Bottom panel shows instantaneous breathing rate (IBR, connected circles) overlaid with the cumulative model estimates (dark blue line). Black circles show IBR values not included in the cumulative model fitting. Tag deployment code is shown on the right, with individual class (SC: Small body size, associated with a calf).


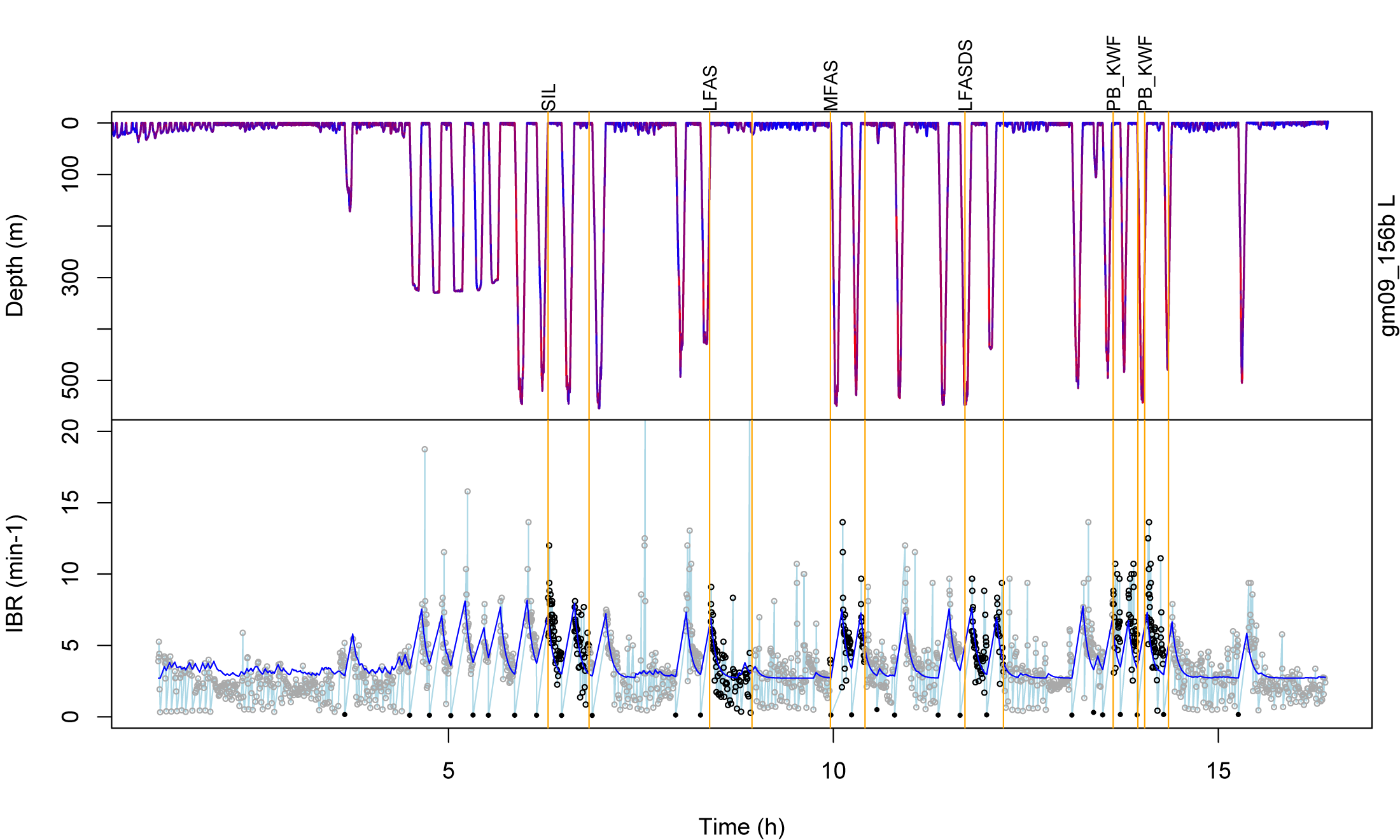


Fig. C7 Top panel shows the dive profile, color-coded by fluke stroke rate (red: higher rate). Orange vertical lines show sound exposure start and end times (SIL: no-sonar approach, LFAS: low-frequency active sonar, MFAS: medium-frequency active sonar, LFASDS: low-frequency active sonar down-sweep signal, PB_KWF: fish-eating killer whale sound playback). Bottom panel shows instantaneous breathing rate (IBR, connected circles) overlaid with the cumulative model estimates (dark blue line). Black circles show IBR values not included in the cumulative model fitting. Tag deployment code is shown on the right, with individual body size class (L: Large).


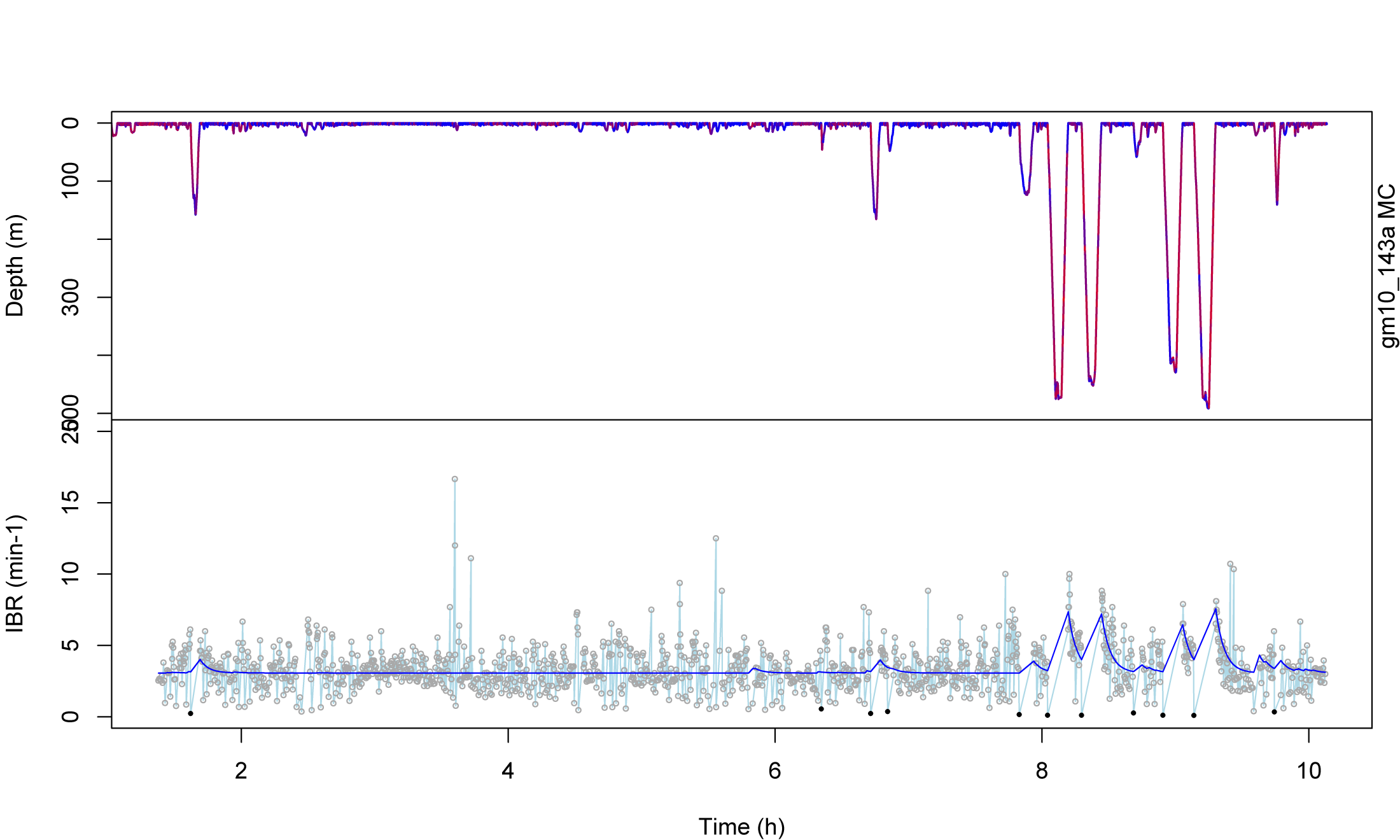


Fig. C8 Top panel shows the dive profile, color-coded by fluke stroke rate (red: higher rate). Black circles show IBR values not included in the cumulative model fitting. Tag deployment code is shown on the right, with individual class (MC: Medium body size, associated with a calf).


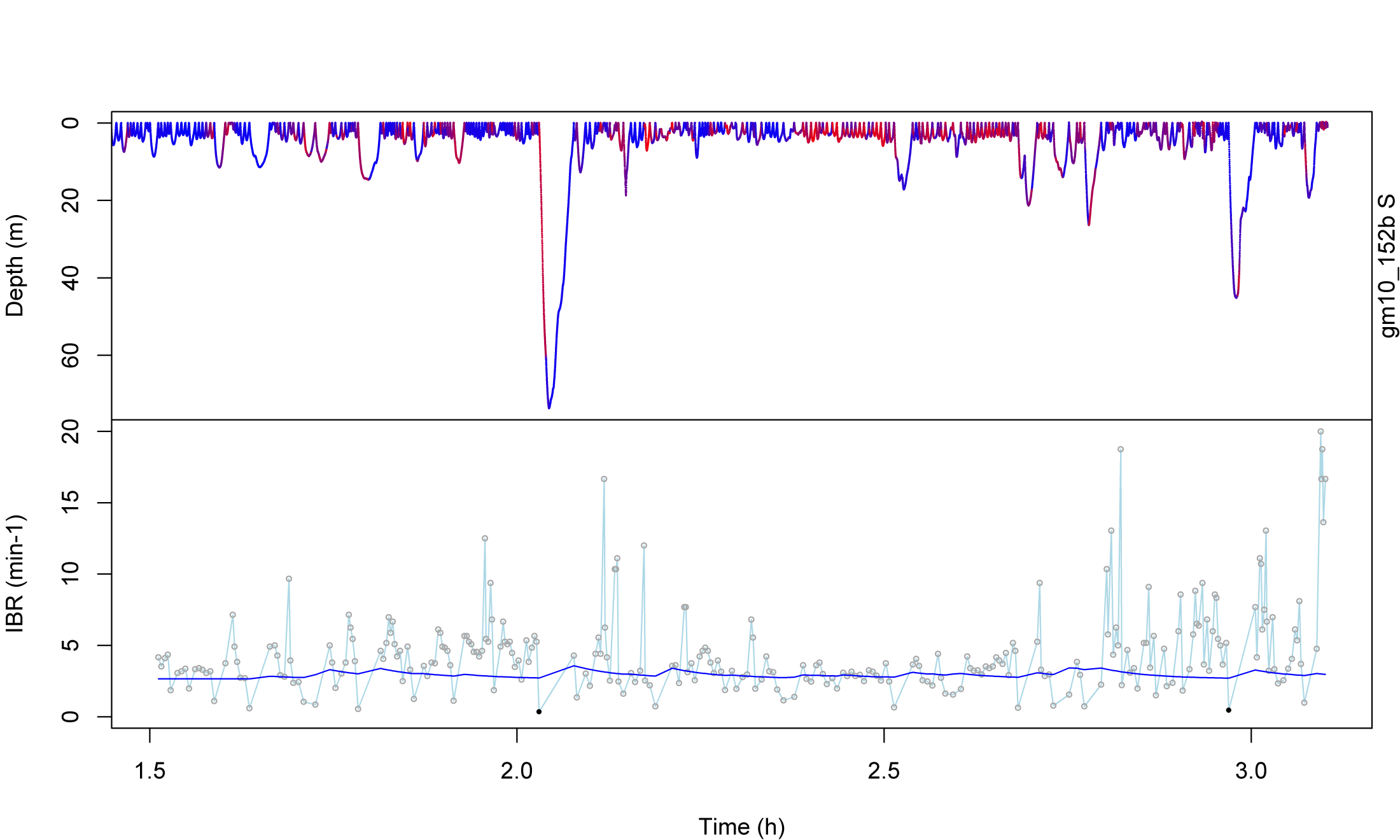


Fig. C9 Top panel shows the dive profile, color-coded by fluke stroke rate (red: higher rate). Bottom panel shows instantaneous breathing rate (IBR, connected circles) overlaid with the cumulative model estimates (dark blue line). Black circles show IBR values not included in the cumulative model fitting. Tag deployment code is shown on the right, with body size class (S: Small).


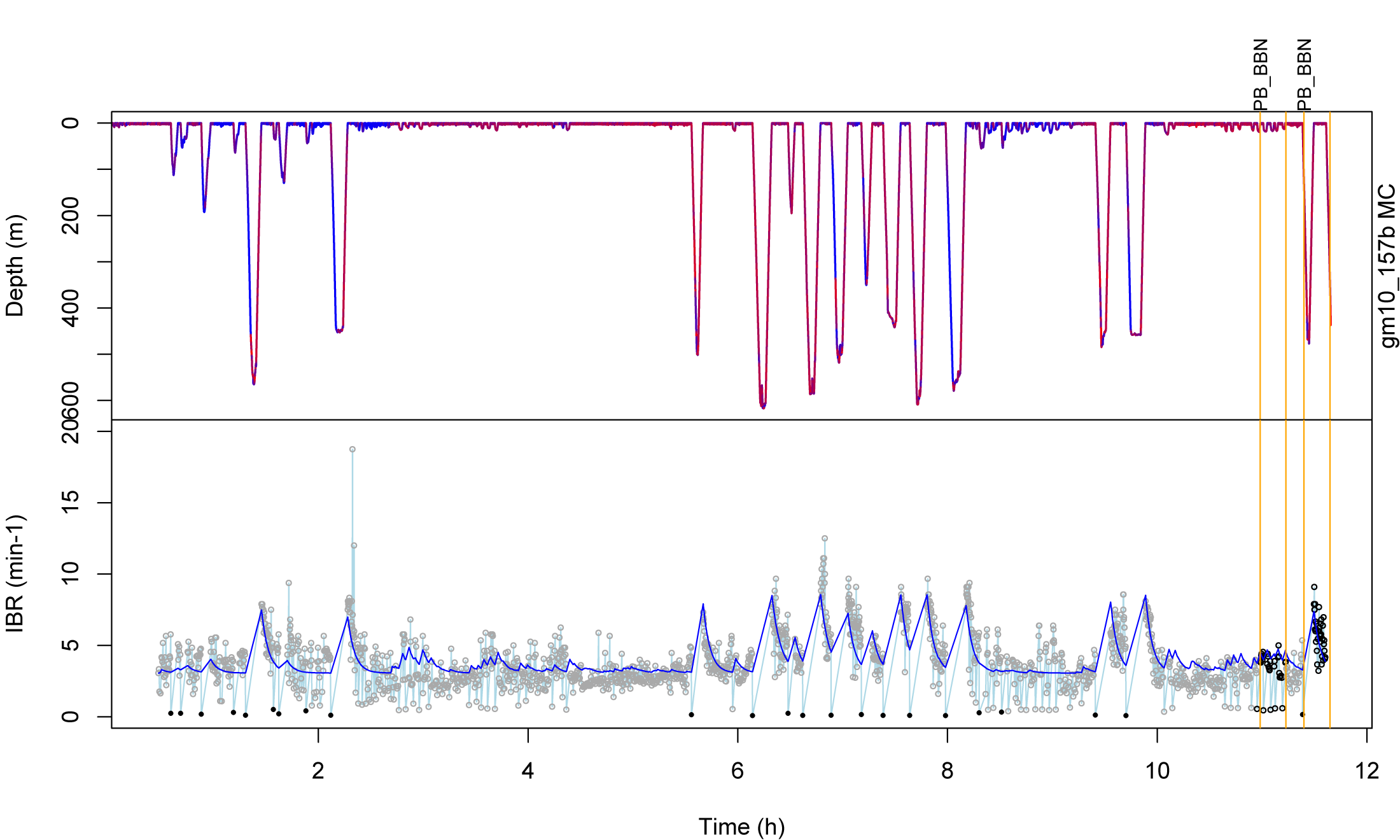


Fig. C10 Top panel shows the dive profile, color-coded by fluke stroke rate (red: higher rate). Orange vertical lines show sound exposure start and end times (PB_BBN: broad-band noise control playback). Bottom panel shows instantaneous breathing rate (IBR, connected circles) overlaid with the cumulative model estimates (dark blue line). Black circles show IBR values not included in the cumulative model fitting. Tag deployment code is shown on the right, with individual class (MC: Medium body size, associated with a calf).


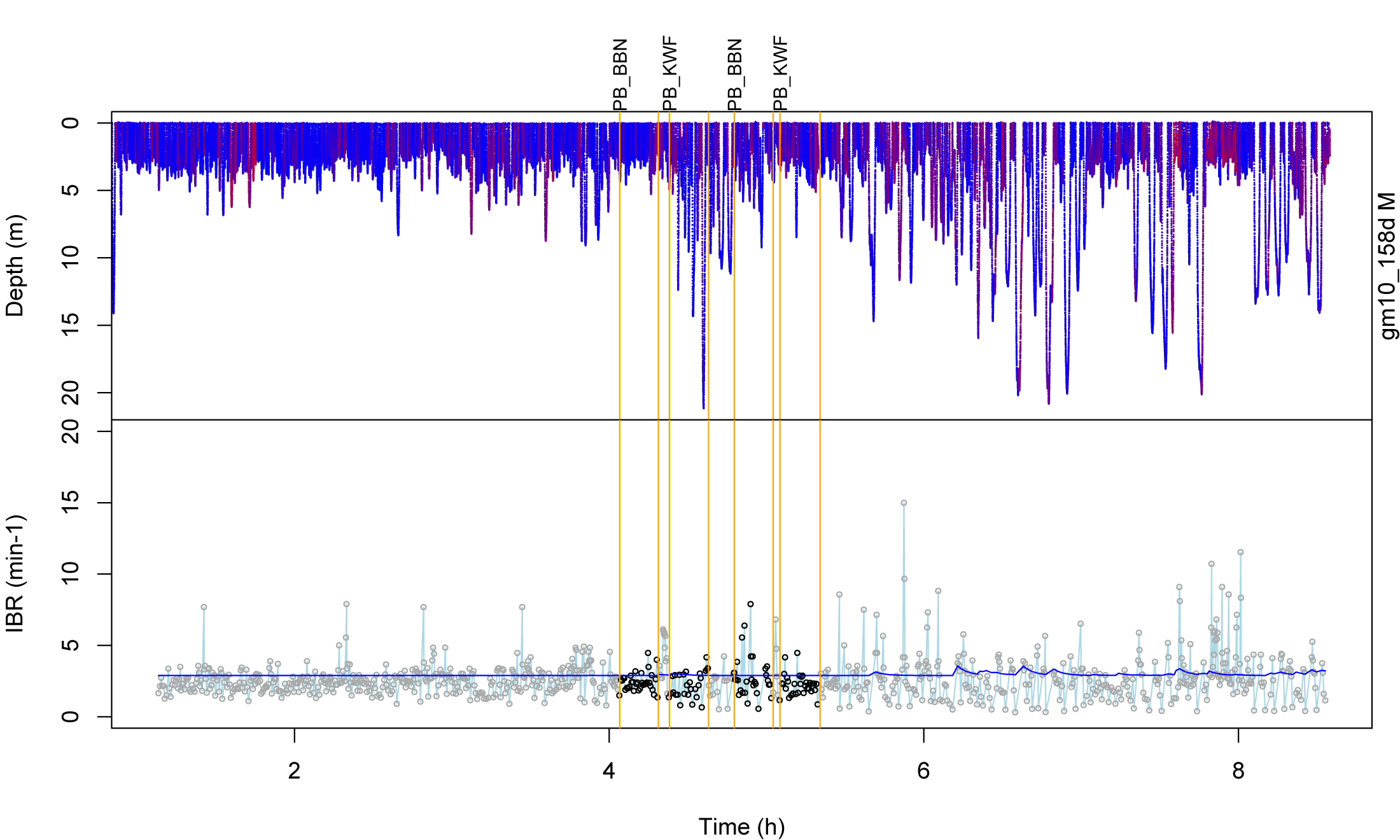


Fig. C11 Top panel shows the dive profile, color-coded by fluke stroke rate (red: higher rate). Orange vertical lines show sound exposure start and end times (PB_KWF: fish-eating killer whale sound playback, PB_BBN: broad-band noise control playback). Bottom panel shows instantaneous breathing rate (IBR, connected circles) overlaid with the cumulative model estimates (dark blue line). Black circles show IBR values not included in the cumulative model fitting. Tag deployment code is shown on the right, with body size class (M: Medium).


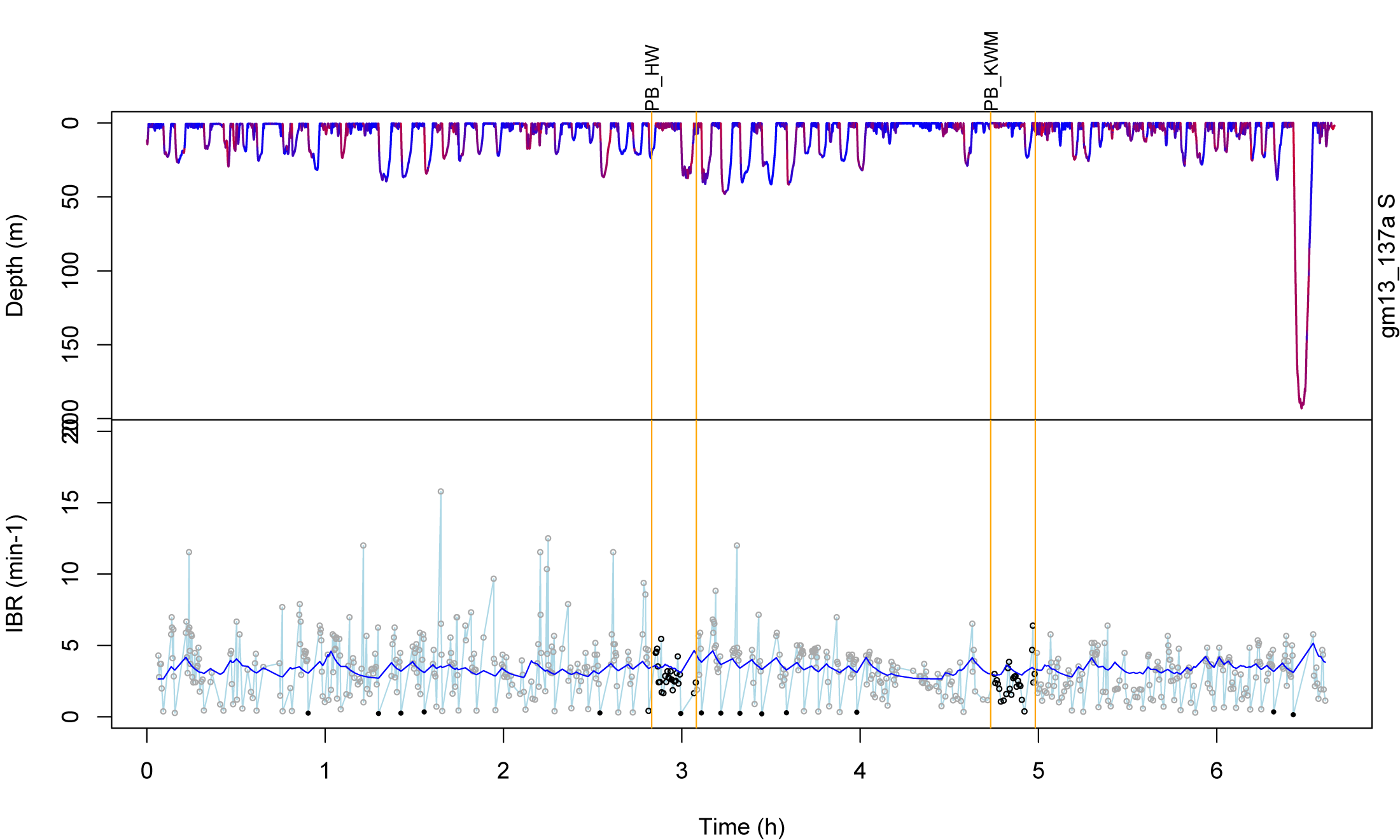


Fig. C12 Top panel shows the dive profile, color-coded by fluke stroke rate (red: higher rate). Orange vertical lines show sound exposure start and end times (PB_HW: playback of humpback whale sounds (a trial control, excluded from all analyses), PB_KWM: mammal-eating killer whale sounds playback). Bottom panel shows instantaneous breathing rate (IBR, connected circles) overlaid with the cumulative model estimates (dark blue line). Black circles show IBR values not included in the cumulative model fitting. Tag deployment code is shown on the right, with body size class (S: Small).


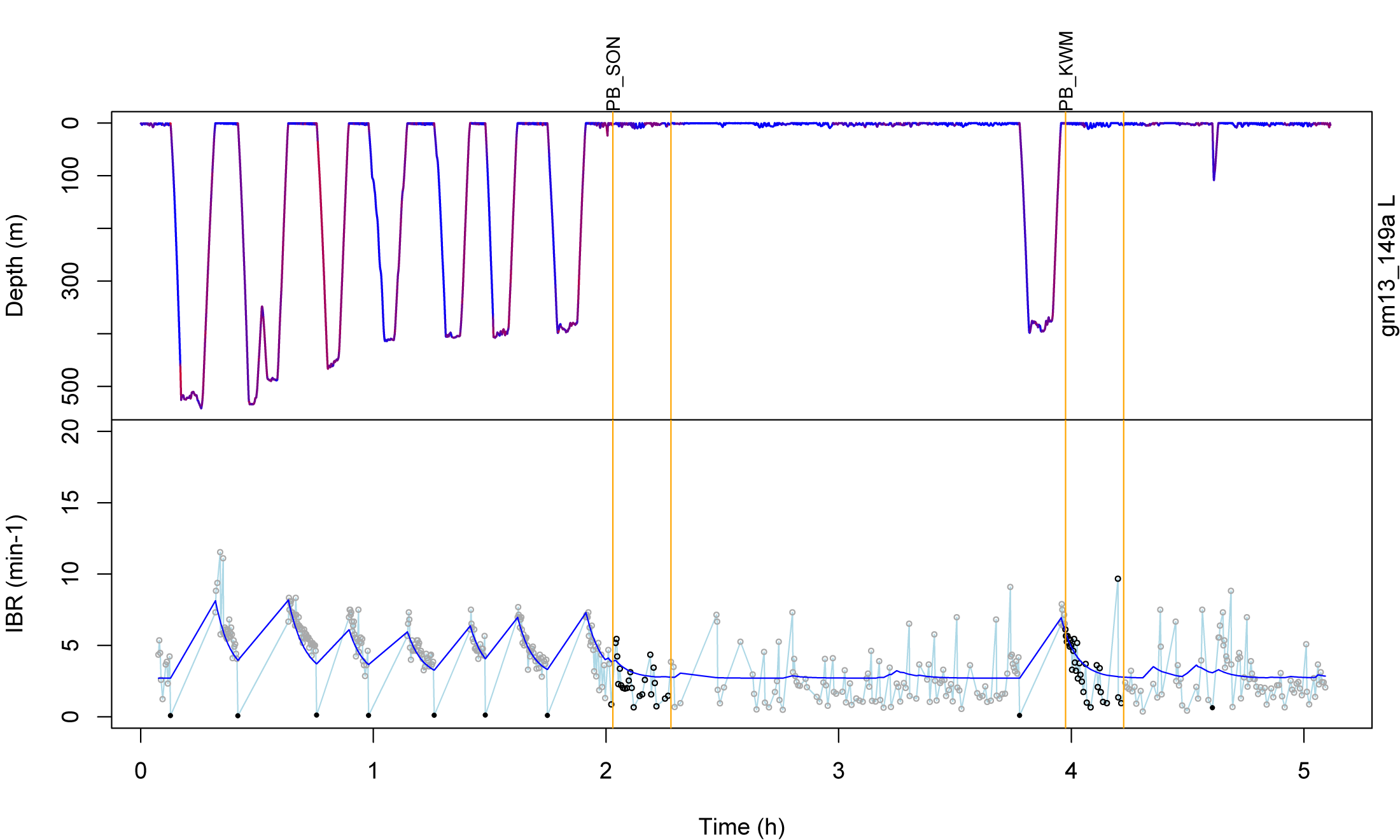


Fig. C13 Top panel shows the dive profile, color-coded by fluke stroke rate (red: higher rate). Orange vertical lines show sound exposure start and end times (PB_SON: playback of 1-2 kHz sonar sounds, PB_KWM: mammal-eating killer whale sounds playback). Bottom panel shows instantaneous breathing rate (IBR, connected circles) overlaid with the cumulative model estimates (dark blue line). Black circles show IBR values not included in the cumulative model fitting. Tag deployment code is shown on the right, with body size class (L: Large).


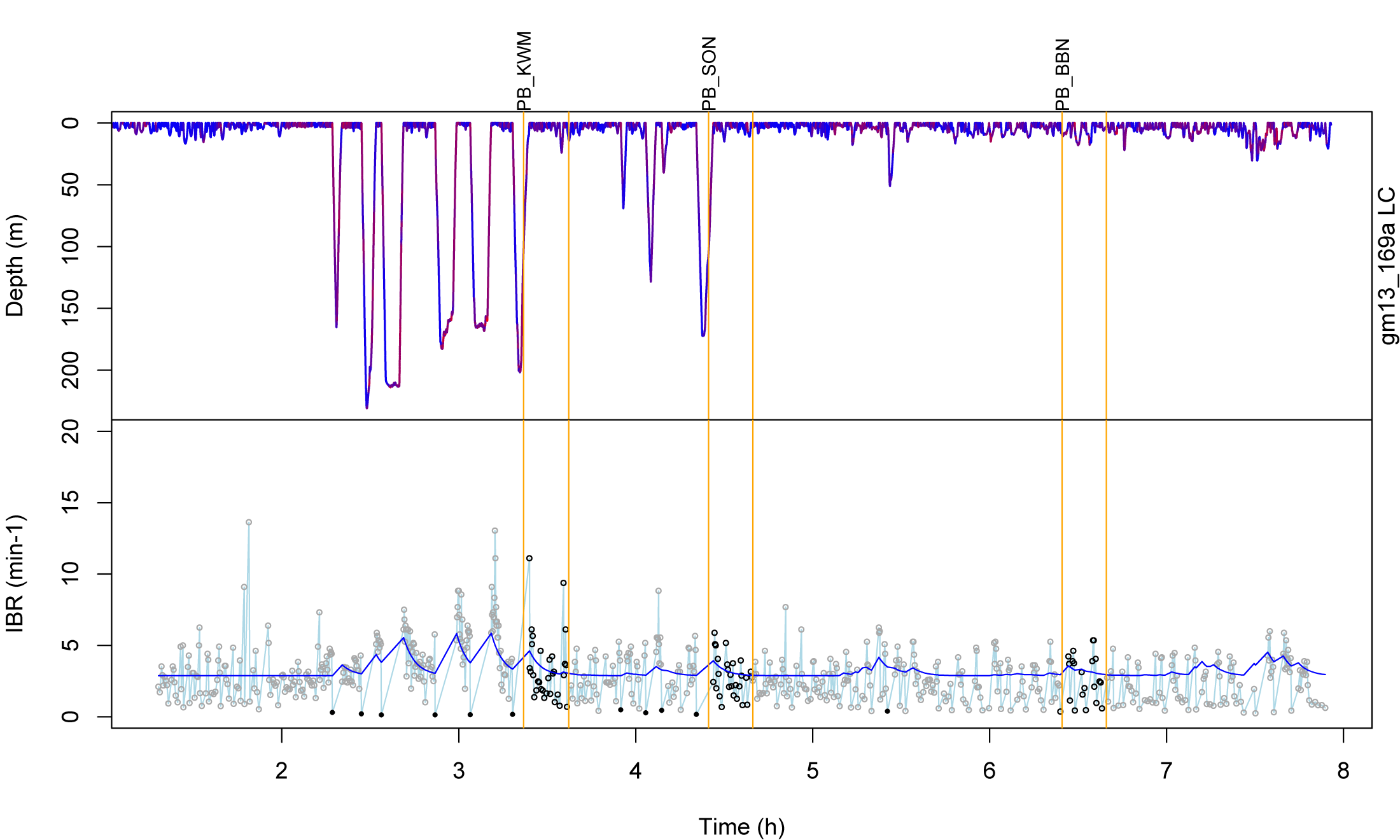


Fig. C13 Top panel shows the dive profile, color-coded by fluke stroke rate (red: higher rate). Orange vertical lines show sound exposure start and end times (PB_KWM: mammal-eating killer whale sounds playback, PB_SON: playback of 1-2 kHz sonar sounds, PB_BBN: broad-band noise control playback). Bottom panel shows instantaneous breathing rate (IBR, connected circles) overlaid with the cumulative model estimates (dark blue line). Black circles show IBR values not included in the cumulative model fitting. Tag deployment code is shown on the right, with individual class (LC: Large body size, associated with a calf).


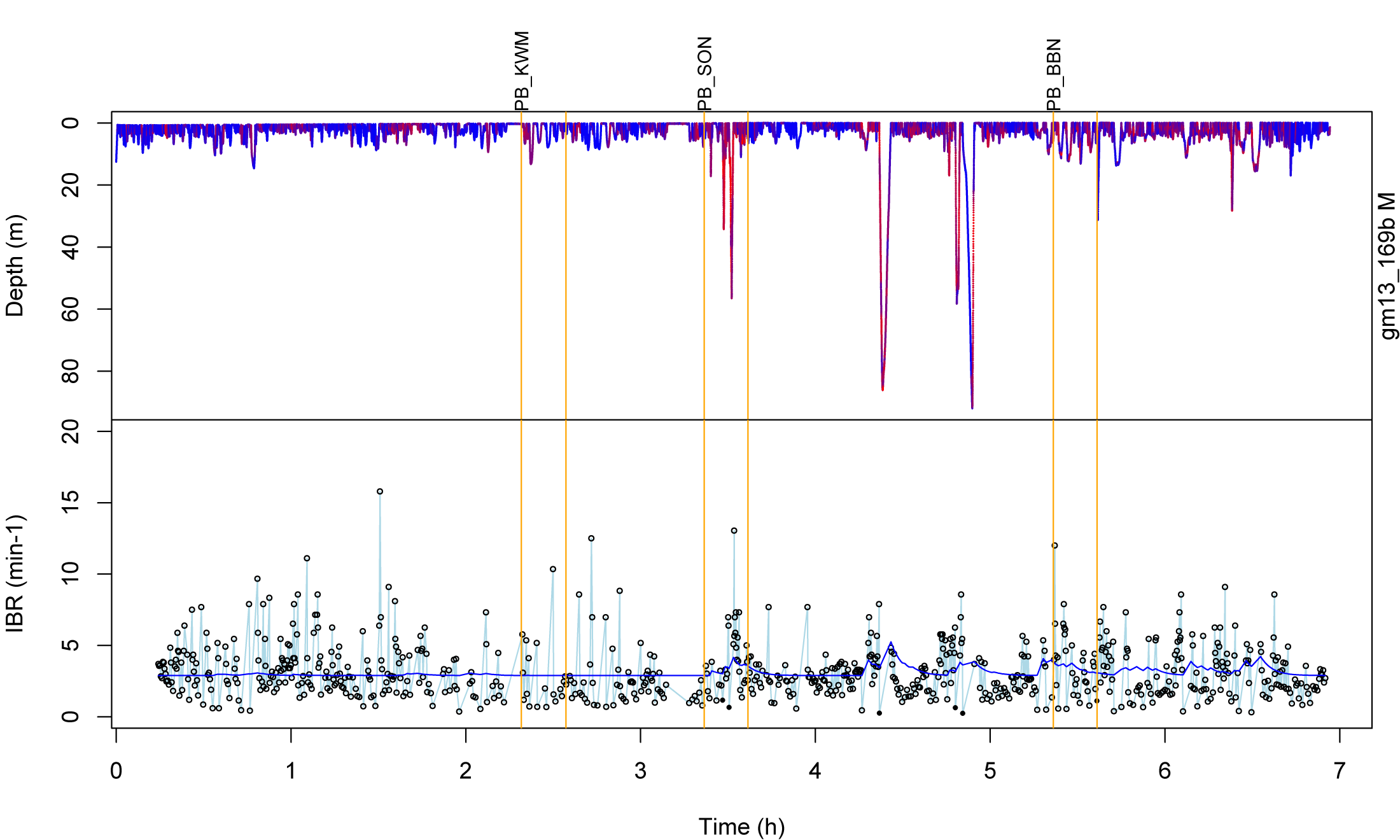


Fig. C15 Top panel shows the dive profile, color-coded by fluke stroke rate (red: higher rate). Orange vertical lines show sound exposure start and end times (PB_SON: playback of 1-2 kHz sonar sounds, PB_KWM: mammal-eating killer whale sounds playback, PB_BBN: broad-band noise control playback). Bottom panel shows instantaneous breathing rate (IBR, connected circles) overlaid with the cumulative model estimates (dark blue line). Black circles show IBR values not included in the cumulative model fitting. Tag deployment code is shown on the right, with body size class (M: Medium).


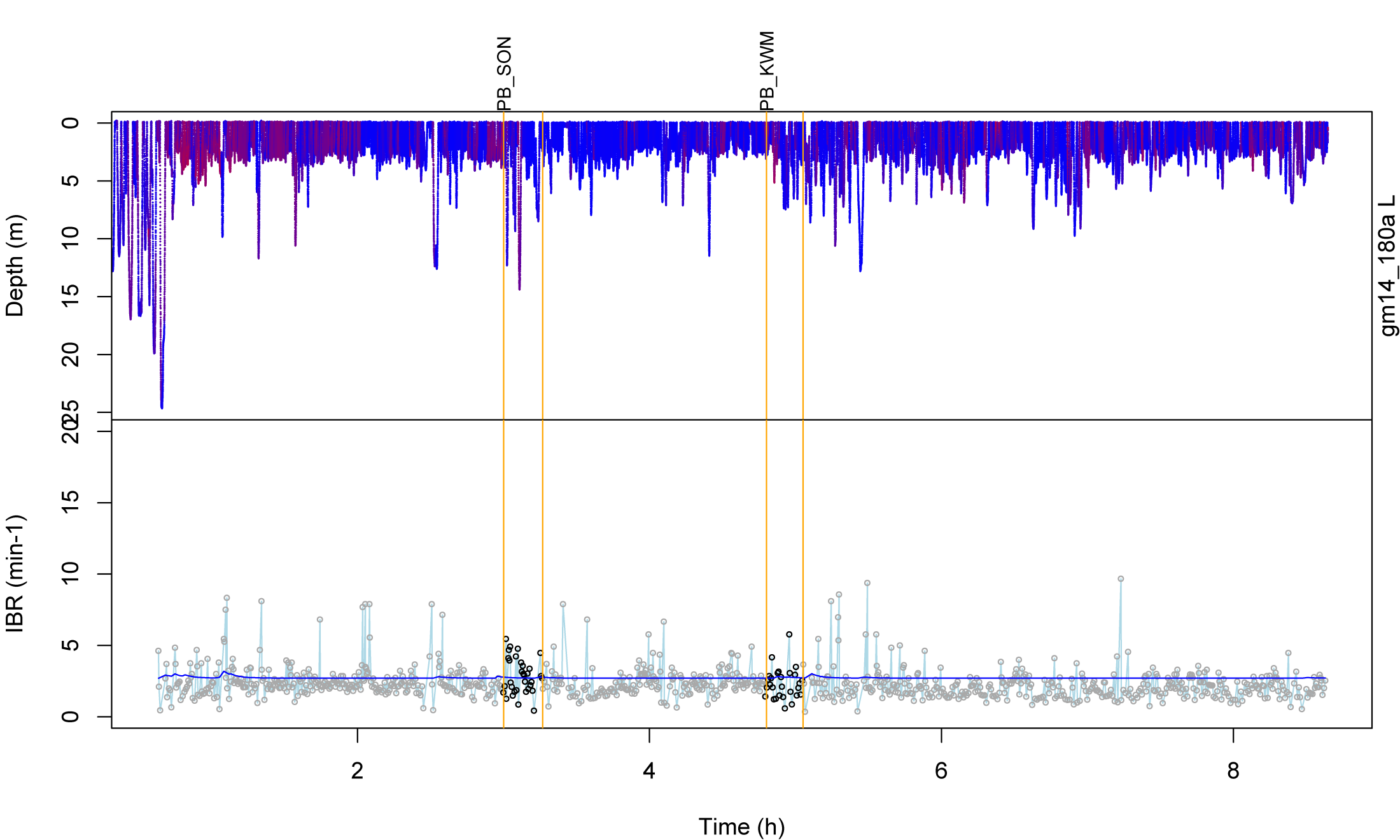


Fig. C16 Top panel shows the dive profile, color-coded by fluke stroke rate (red: higher rate). Orange vertical lines show sound exposure start and end times (PB_SON: playback of 1-2 kHz sonar sounds, PB_KWM: mammal-eating killer whale sounds playback). Bottom panel shows instantaneous breathing rate (IBR, connected circles) overlaid with the cumulative model estimates (dark blue line). Black circles show IBR values not included in the cumulative model fitting. Tag deployment code is shown on the right, with body size class (L: large).


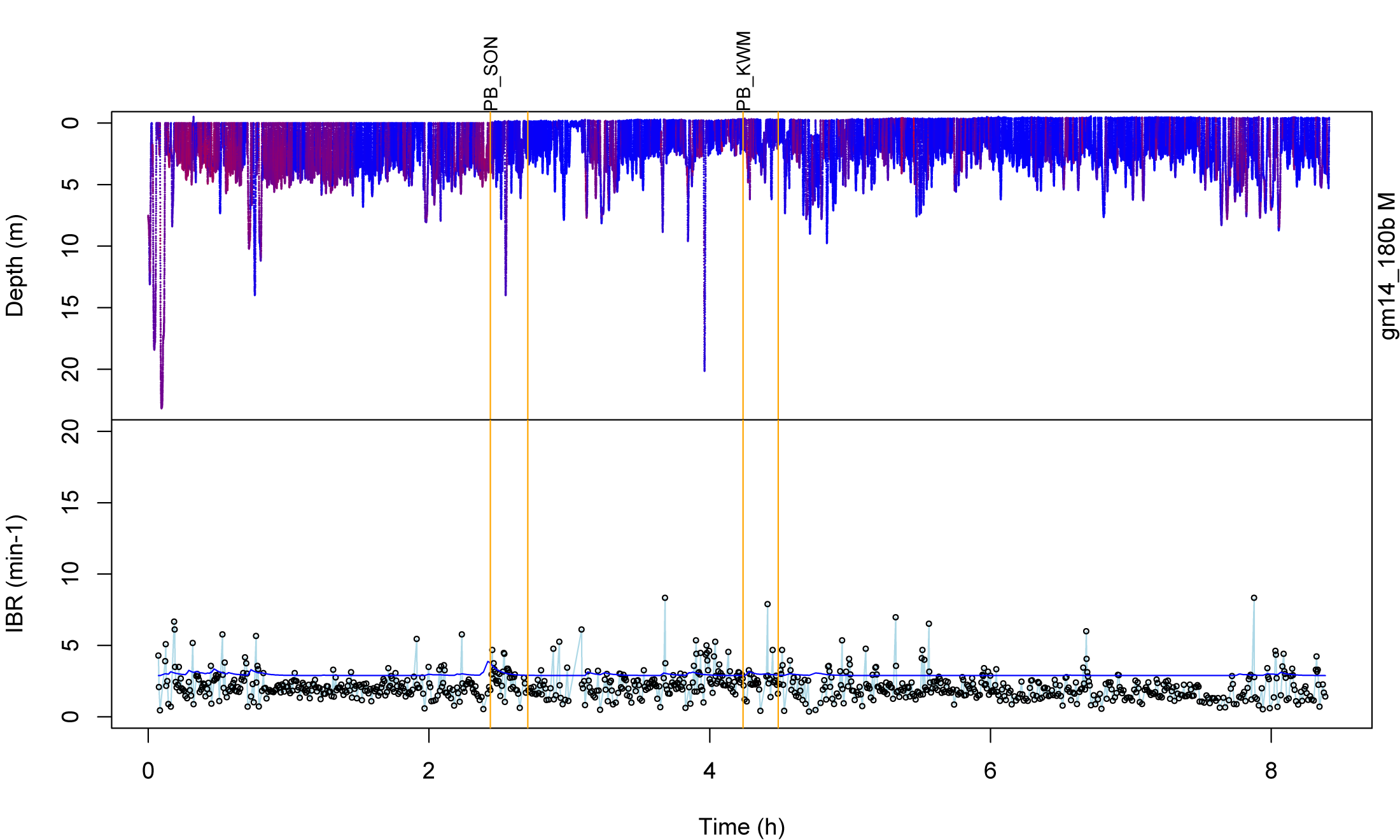


Fig. C17 Top panel shows the dive profile, color-coded by fluke stroke rate (red: higher rate). Orange vertical lines show sound exposure start and end times (PB_SON: playback of 1-2 kHz sonar sounds, PB_KWM: mammal-eating killer whale sounds playback). Bottom panel shows instantaneous breathing rate (IBR, connected circles) overlaid with the cumulative model estimates (dark blue line). Black circles show IBR values not included in the cumulative model fitting. Tag deployment code is shown on the right, with body size class (M: medium).
