# Supplementary material for: Breathing Patterns Indicate Cost of Exercise During Diving and Response to Experimental Sound Exposures in Long-Finned Pilot Whales
Source: Front Physiol. 2018 Oct 25;9:1462. doi: 10.3389/fphys.2018.01462 (PMC6232938; doi:10.3389/fphys.2018.01462)
Supplement: Supplementary file 4 [file Table_4.docx]

Data report

Breathing patterns indicate cost of exercise during diving and response to experimental sound exposures in long-finned pilot whales

Saana Isojunno^*^, Kagari Aoki, Charlotte Curé, Petter H. Kvadsheim, Patrick J. O. Miller

*** Correspondence:** Saana Isojunno: si66@st-andrews.ac.uk

**
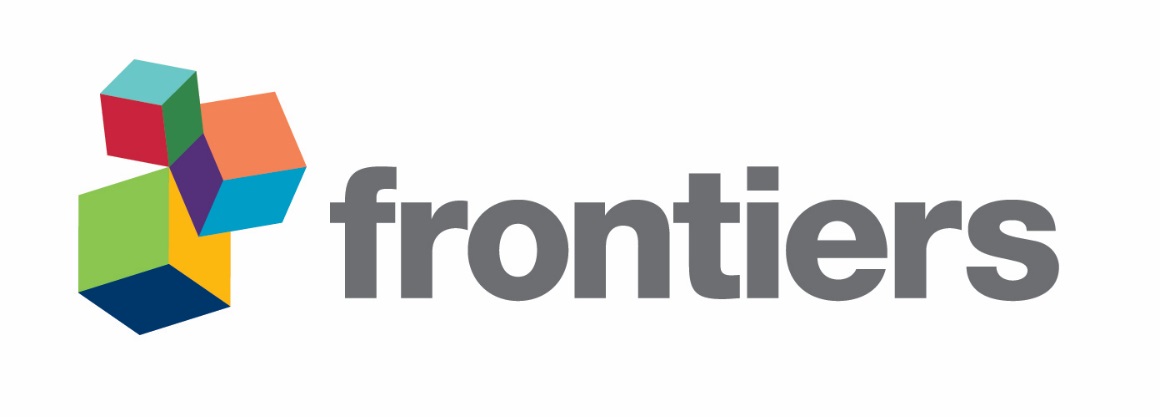
**

Please find enclosed zip file containing all the data and R-code required to conduct the analyses of this manuscript. The data are stored as csv-files and one .Rd file. Rd files can be opened in R using the load() command. The R-code is stored as .R files, which can be opened as a script in R, or using word processing software such as notepad.

The .Rd files containing dive profile and pitch data for each tag deployment were too large (185 MB) to be included as supplementary materials, but can be downloaded from [this dropbox link](https://www.dropbox.com/sh/rat31si6g828y6v/AADD_ibavFHbyFLqpmZKmpr-a?dl=0).

The csv-files contain data for each individual (indsummary.csv), for each experiment (etab.csv) and for each dive/inter-breath interval (IBI) (divedata.csv). The ‘diveprofiles.Rd’ dataset contains depth and pitch data for each individual at 5 Hz (stored as a list). Please see the table below for further details about each data variable.

To run the analysis, place the contents of the zip file into your working directory in R. Run each R script sequentially, from Step 1 to Step 6. The R-script for Step 4 can be run either using the provided model fits (Step4_Model_3_fit_(1).Rd, …, Step4_Model_3_fit_(8).Rd), or re-fitting the models. The latter option will be considerably slower.

| Data set | Column name | Measurement | Notes |
| --- | --- | --- | --- |
| indsummary | ind | Tag id | Tag deployment identifier (species, year, Julian date, a, b …) |
|  | tagstart_str | Tag start time | Tag recording start time (GMT) |
|  | flukefs_m, flukefs_l, flukefs_u | Mean, lower and upper fluke stroke frequency (Hz) | Fluke stroke frequencies measured from periodograms for each tag record |
|  | pitch_filter | Filtering frequency for pitch (Hz) | High pass filter used to filter pitch data before detection of fluke strokes |
|  | Calf | Calf association | 1 – calf associated, 0 – calf not associated |
|  | Size | Body size class | Body size class based on field notes and fin width (5 classes) |
|  | Size2 | Body size class 2 | Body size class with three classed, combining ‘Very small’ and ‘Small’ as ‘Small’, and ‘Medium’ and ‘Medium-Large’ to ‘Medium’ |
| etab | tagid | Tag id | Tag deployment identifier (species, year, Julian date) |
|  | focal1 | Focal tag id | Focal tag id – the whale that was visually tracked |
|  | focal2 | Non-focal tag id | Non-focal tag id - whale deployed in the vicinity of the Focal whale, but was not the focus of visual trakcing |
|  | Session | Experimental session | MFAS=6-7 kHz sonar, LFAS=1-2 kHz sonar, SIL=no-sonar control approach, PB_KWF/PB_KWM=playback of fish-eating/mammal-eating killer whale sounds, PB_BBN=playback of broad band noise (negative control), PB_HW=playback of humpback whale sounds (a pilot study, excluded from analysis) |
|  | GMT_start, GMT_end | Start and end time (GMT) |  |
| divedata | ind | Tag id | Tag deployment identifier (species, year, Julian date, a, b, …) |
|  | dive.sfromtot | Start time (s) | Start time of the IBI, seconds from tag recording start time |
|  | dive.GMTtime | Start time (GMT) | Start time of the IBI, in GMT |
|  | dive.end | End time (s) | End time of the IBI, seconds from tag recording start time |
|  | dive.dur | Dive duration (s) |  |
|  | dive.depth | Dive depth (m) | Maximum depth of the IBI |
|  | surf.end | Surface end (s) | End time of the following surface interval, seconds from tag recording start time |
|  | surf.dur | Surface interval (s) | Duration of the following surface interval |
|  | ind.type | Individual type | Tagged whale body size, levels: Small (S), Medium (M), Large (L), followed by calf association (C), if present |
|  | ind.small, ind.med, ind.large | Individual body size class | Presence/absence variables for individual body size (small, medium, large) |
|  | ind.TBM | Individual body mass (kg) | Individual body mass estimated from fundamental fluke stroke frequency for each tag |
|  | dive.fluken | Number of fluke strokes | Counted for during the dive (dive.sfromtot – dive.end) |
|  | pch | Symbol shape | Used to plot each individual type as different symbol |
|  | Ind.col | Individual colour | Used to plot each tag deployment as different colour |
|  | Session | Exposure session | What exposure session the dive belongs to – based on majority of dive time, except for pre-exposure baseline where the whole dive must be not exposed. MFAS=6-7 kHz sonar, LFAS=1-2 kHz sonar, SIL=no-sonar control approach, PB_KWF/PB_KWM=playback of fish-eating/mammal-eating killer whale sounds, PB_BBN=playback of broad band noise (negative control), PB_HW=playback of humpback whale sounds (a pilot study, excluded from analysis) |
|  | RL_max | Max received level (dB re 1 μPa m) | Maximum sound pressure (SPL) level during the dive |
|  | dive.SIL, dive.LFAS, dive.MFAS, dive.PB_BBN, dive.PB_KWF, dive.PB_KWM, dive.PB_SON, dive.PB_HW | Exposure session coverage (proportion) | Proportion of dive time falling into each exposure session |
|  | tsince.SIL, tsince.LFAS, tsince.MFAS, tsince.PB_BBN, tsince.PB_KWF, tsince.PB_KWM, tsince.PB_SON, tsince.PB_HW | Time since exposure (min) | Time since exposure session ends, at the start of the dive. Set to 30 min before exposure. |
|  |  |  |  |
| Dive profiles | whaleList | List of tag deployments | Tag deployment identifier (species, year, Julian date, a, b, …) |
|  | tempList | List of data, one element for each tag deployment |  |
|  | datetime | Time (GMT) |  |
|  | sfromtot | Time (s) | Seconds from tag recording start |
|  | whale | Tag id | Tag deployment identifier |
|  | p | Depth (m) |  |
|  | pitch | Pitch (radians) | Raw pitch data |
|  | pitch_filt | Filtered pitch (rad) | Filtered pitch data |
